# Counterfactual Reasoning Underlies the Learning of Priors in Decision Making

**DOI:** 10.1016/j.neuron.2018.07.035

**Published:** 2018-09-05

**Authors:** Ariel Zylberberg, Daniel M. Wolpert, Michael N. Shadlen

**Affiliations:** 1 Department of Neuroscience, Zuckerman Mind Brain Behavior Institute, Columbia University, New York, NY 10027, USA; 2 Howard Hughes Medical Institute, Columbia University, New York, NY 10027, USA; 3 Computational and Biological Learning Laboratory, Department of Engineering, Cambridge University, Cambridge CB2 1PZ, UK; 4 Kavli Institute, Columbia University, New York, NY 10027, USA

## Abstract

Accurate decisions require knowledge of prior probabilities (e.g., prevalence or base rate), but it is unclear how prior probabilities are learned in the absence of a teacher. We hypothesized that humans could learn base rates from experience making decisions, even without feedback. Participants made difficult decisions about the direction of dynamic random dot motion. Across blocks of 15–42 trials, the base rate favoring left or right varied. Participants were not informed of the base rate or choice accuracy, yet they gradually biased their choices and thereby increased accuracy and confidence in their decisions. They achieved this by updating knowledge of base rate after each decision, using a counterfactual representation of confidence that simulates a neutral prior. The strategy is consistent with Bayesian updating of belief and suggests that humans represent both true confidence, which incorporates the evolving belief of the prior, and counterfactual confidence, which discounts the prior.

## Highlights

• People can learn base rates without feedback and apply them to make better decisions
• The estimate of base rate is updated based on the confidence in each decision
• The form of confidence used is counterfactual, as if the base rate were uninformative
• The study extends the Bayesian framework from choice to prior probability estimation

## Teaser

Zylberberg et al. show that human decision makers can learn environmental biases from sequences of difficult decisions, without feedback about accuracy, by calculating the belief that the decisions would have been correct in an unbiased environment—a form of counterfactual confidence.

## Introduction

Accurate decision making relies on both evidence bearing on the choice at hand and prior knowledge about the statistical regularities bearing on the possible options. In some instances, we learn these regularities through education (e.g., disease prevalence based on decisions of experts), but more often such knowledge is acquired over time through decisions we make ourselves. This poses a problem because without an omniscient teacher, our decisions can be inaccurate, which limits our ability to update our beliefs. The problem is compounded because the decisions we make may be affected by our evolving belief of the statistical regularities, thereby biasing our decisions, which could in turn affect how we update our belief about the regularities.

As an example, consider you are working on a production line determining whether tomatoes arriving one at a time on a conveyor belt are ripe and ready to ship or unripe and need to be held back. Some are obviously red and ripe and others are clearly green and unripe, but there are others on the border that will be harder to judge. Each crate comes from a different supplier and will likely have a different proportion of unripe tomatoes. If you knew each crate’s proportion of unripe tomatoes (the base rate), it would help you sort the tomatoes. This is an example in which one is learning a prior (base rate) without a teacher or confirmation about ground truth.

One possibility is that one would ignore the base rate in this setting, but that would be unwise when confronted with an ambiguous tomato. It makes more sense to estimate the base rate from one’s experience. One could simply use the proportion of decisions of unripe as the estimate of the base rate. However, this would give equal weighting to tomatoes judged as clearly unripe and those on the borderline. An alternative might be to use a measure of confidence in determining the base rate, but this invites another challenge. If one uses the base rate to decide ripeness, this will affect the tally and potentially bias the estimate of the base rate itself.

This simple example illustrates the complexity of doing inference in a world in which one is simultaneously learning a model and applying it. It arises in medical decision making (Medin and Edelson, 1988, Goldberg, 1970), weather prediction (Knowlton et al., 1994, Yang and Shadlen, 2007), and other inference problems that benefit from experience but for which feedback about ground truth is unavailable on a useful timescale. It has been shown that people are able to estimate base rates from a sequence of observations (Phillips and Edwards, 1966, Estes, 1972, Peterson and Beach, 1967) to develop a bias that serves as prior knowledge in subsequent interactions with the environment (Anderson and Carpenter, 2006, Manis et al., 1980) and even use these observations to infer changes in the state of the environment (Summerfield et al., 2011, Behrens et al., 2007, Meyniel et al., 2015, Purcell and Kiani, 2016, Nassar et al., 2010, Rapoport, 1964, Carpenter and Williams, 1995). In these cases, the observations that inform the prior are clearly discernible (e.g., target reached/not reached) or are accompanied by explicit feedback. For example, if the sorter were to taste each tomato, the ground truth could be known, but there would be no tomatoes shipped. We hypothesized that in the absence of explicit feedback, decision confidence guides the acquisition of prior probability and does so in accordance with Bayesian updating. We build on recent progress in the understanding of confidence in simple perceptual decisions.

To study the role of confidence in the acquisition of prior probability, we designed a task in which human participants made a sequence of binary decisions in the presence of a concealed base rate that favored one of the alternatives. The decisions involved judging the direction of motion of a set of randomly moving dots, which were made without feedback. The base rate was constant within a block of trials but randomly varied from one block to another. Crucially, the base rate was not known to the participant. As the participants made more decisions, the influence of base rate on choice and confidence increased, which was reflected both in the decision about the direction of motion and in an explicit report about the bias of the block. A bounded evidence accumulation model explained the decisions about motion by incorporating an estimate of the base rate in the accumulation. In turn, a probability distribution over base rates was updated based on the likelihood that the motion was rightward or leftward—what we term counterfactual posterior probability or counterfactual confidence—under the fictitious supposition that the alternatives were equally likely (cf. Bernardo, 1979). The model predicted the dynamics of belief about the direction bias over the block. The findings expose a role for counterfactual confidence in belief updating, suggesting that the brain maintains probabilistic representations over decision hierarchies and timescales: direction over one trial and bias over many trials. Further, these probabilities are accessible for explicit reporting.

## Results

Three participants were presented with a dynamic display of random dots of variable duration and had to decide whether the net direction of motion was rightward or leftward. Within a block of 15 to 42 trials, one direction of motion (left or right) was more likely, but which direction was more likely (and by how much) was unknown to the participant. The difficulty of the decision was controlled by three factors: strength of motion, stimulus duration, and bias strength (i.e., base rate). Motion strength was controlled by the probability (termed motion coherence [*c*]) that a dot is displaced in motion as opposed to randomly. Stimulus duration was sampled from a truncated exponential distribution (range: [0.1, 0.9] s). Bias strength was controlled by the probability that the motion direction would be rightward, termed *B*, which was selected randomly on each block from six possible values ranging from 0% to 100% in steps of 20%. Participants knew the possible values of *B*, and that they were equally likely, but were not told which one applied to the current block.

**Figure 1:**
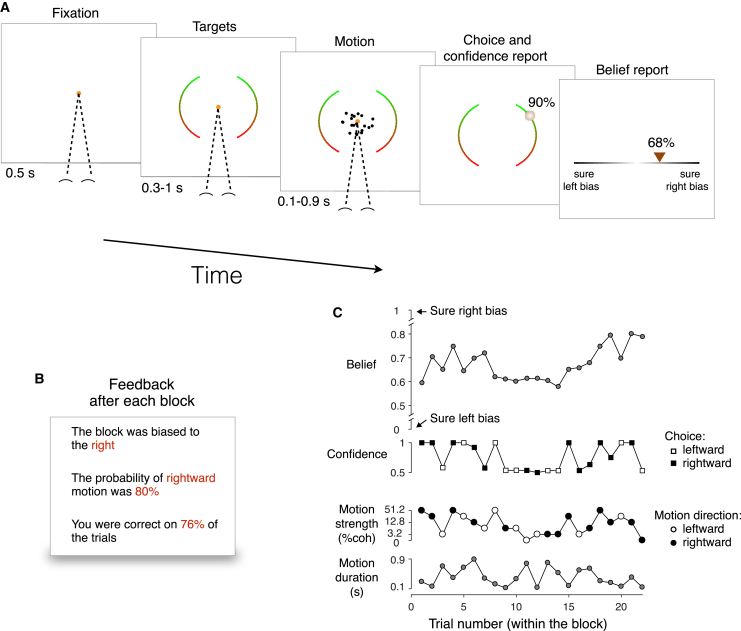
Motion Discrimination Task Each block of 15 to 42 trials was assigned one of six possible base rates: the prior probability that the motion is rightward. In all blocks, the subject discriminated the direction of random dot motion. No feedback about individual decisions or the base rate was provided until the end of the block. (A) Sequence of events within a trial. After fixation and a random delay, random dot motion was displayed for 0.1–0.9 s (truncated exponential). Subjects then positioned a cursor on the left or right arcs to indicate both choice (left versus right) and confidence (100% certainty [top] to guessing [bottom]). After the motion decision, the subjects reported whether they believed that the block had a rightward or leftward bias (placing the cursor in the left or right half of the line), together with the confidence in this belief (from unsure at the center to certainty at the extremes). (B) Example of the feedback display provided at the end of a block. (C) Example of a sequence of trials within a block. Lower two graphs show variables controlled by the computer: motion strength, direction, and duration. Upper two graphs show the subject’s reports: direction choice, confidence that the choice was correct, and belief that the base rate favored rightward. An example of the task is shown in Video S1. Video S1. Motion Discrimination Task, Related to Figure 1Example of the sequence of events within a short block of trials.

Participants made three responses in each trial. They first reported the perceived direction of motion and the confidence that this decision was correct (Figure 1A, Choice and confidence report). They then reported whether they considered the block to have a right or left bias and the confidence in this judgment (Figure 1A, Belief report). To avoid confusion, we refer to this type of confidence as “belief,” an estimate of the probability that the block has a rightward bias (scale 0 to 1). Participants received no feedback about the accuracy of their decisions during a block of trials. Only after completing a block were they told which direction was the most probable, the strength of the bias in this direction (either 60%, 80%, or 100%), and the proportion of trials in which they responded correctly (Figure 1B).

Figure 1C shows a typical sequence of events and reports that transpire in a single block in the experiment. The bottom two rows show the particular sequence of 22 trials, each associated with motion stimulus of some strength, direction, and duration. The upper two rows show the corresponding behavioral reports: direction choice, confidence in the choice, and belief about bias of the block. In this block, the participant was correct on most of the trials, with a confidence that was strongly correlated with motion strength. At the beginning of the block, the belief was in the region of high uncertainty (∼0.5) and evolved to greater certainty later in the block. As a glimpse into what we will appreciate in greater detail later, it can be seen that decisions made with high confidence are usually followed by larger changes in belief than decisions made with low confidence. This is evident for trials 9 to 14, which shows a sequence of low-confidence decisions accompanied by subtle changes in belief. The example provides an intuition for the inference problem the participant confronts on each short block of trials.

We next describe the main effect of the base rate on the direction choices, the confidence in these choices and the belief that the block is biased to the left or right. We then develop a theory to explain the way the direction decisions inform belief and the way belief biases those choices. Finally, we use this theory to predict the time course (evolution) of this belief. We contrast this theory with alternatives.

### Effect of Base Rate on Choice, Confidence, and Belief

**Figure 2:**
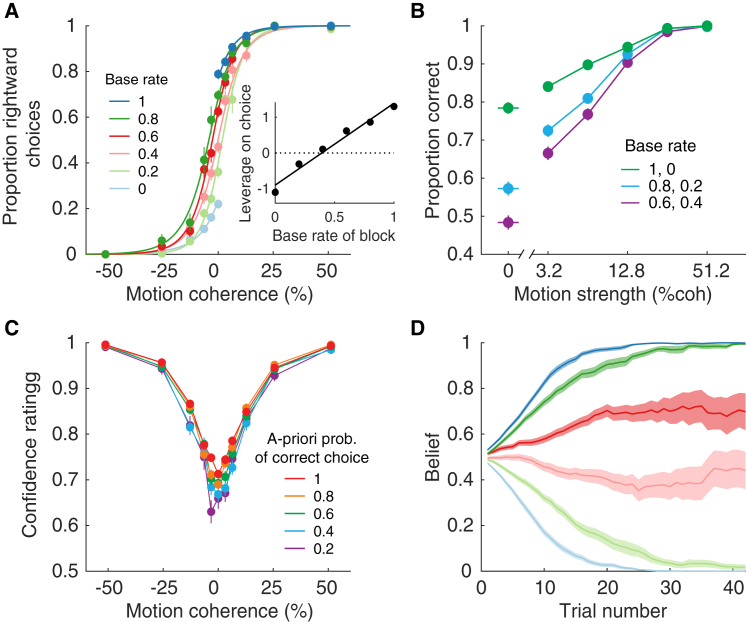
Behavior Was Influenced by the Base Rate (A) Proportion of trials on which the participants reported rightward motion as a function of motion coherence. Data (symbols) are shown separately for the six levels of base rate, from 0 (leftward was the correct choice for all trials in the block) to 1 (all rightward), combining all stimulus durations and trials in the block. The solid lines are fits of a logistic regression model (Equation 19). The shift in the psychometric functions indicate that participants choices were influenced by the base rate. Inset shows the magnitude of the bias ($\beta_{3}$, Equation 19) against the actual base rate (error bars are SE; most are smaller than the points; solid line is least-squares fit). (B) Effect of motion strength and base rate on choice accuracy (same data as in A).The base rates (color) are combined by degree of informativeness. (C) Average confidence reported on correct trials as a function of the motion coherence. The *a priori* probability of correct (color) is an expression of the base rate relative to the choice that is made (see text). Error bars represent SE across trials. (D) Average belief as a function of trial number within a block for the six different biases. Same color convention as in (A). A belief of 0 or 1 indicates full certainty that the block was biased to the left or right, respectively. Shading indicates SEM. Data are combined across all participants. The same analyses for each participant are shown in Figure S1, and the distributions of confidence and belief for each subject are shown in Figure S2.

Throughout each block, choices were governed by the strength and direction of random dot motion. Figure 2A shows the proportion of rightward choices as a function of stimulus strength, combining data from all trials sharing the same base rate (color). When the base rate strongly favored rightward or leftward (1 or 0, respectively), nearly all of the choices were consistent with the base rate. At the intermediate base rates, the shift was less pronounced. To capture the effect in a model-free way, we performed logistic regression (solid curves) and estimated the choice bias (Equation 19, STAR Methods). As shown by the inset, the subjects clearly internalized the base rate during the block (Equation 19; $p<10^{-8}$; likelihood ratio test; $H_{0}$: all $\beta_{3}=0$). That is, for the same motion strength, subjects were more likely to choose the direction consistent with the base rate of the block. We show combined data from all subjects (individual subjects are presented in Figure S1). It is clear from these observations that the subjects acquire knowledge about the base rate of the block despite the absence of feedback about whether their decisions were correct. Knowledge of the base rate should improve the performance on the direction task. This is clearly supported by Figure 2B, which shows accuracy as a function of motion strength (|c|), and groups base rates of similar strength. We adduce from these observations that participants incorporated knowledge of the base rate to bias and improve their decisions.

Subjects furnished two additional reports—confidence and belief—which indicate that they formed an impression of the prior probability about direction over the course of a block. The confidence reports associated with each decision were clearly influenced by the base rate. Figure 2C shows the confidence ratings for correct choices split by the *a priori* probability that the direction of motion supports the choice that the participant made (correct choices only). For example, the *a priori* probability of 0.8 groups together right choices with rightward motion (positive coherence) in blocks with base rate of 0.8 and left choices with leftward motion (negative coherence) in blocks with base rate of 0.2. Two features of the confidence ratings stand out. The subjects were least confident when the motion was weak (coherence near zero; Equation 20; $p<10^{-8}$, t test, $H_{0}:\beta_{1}=0$). Moreover, confidence was higher when the base rate was more informative (Equation 20; $p<10^{-8}$, t test, $H_{0}:\beta_{2}=0$). This is important because it implies that subjects were not merely choosing one direction more often (e.g., out of habit) but that they incorporated knowledge of the base rate to reduce uncertainty in the decision.

The second report was the belief that the base rate of the block favored right or left. This “belief” evolved gradually during the course of the block (Figure 2D). Note that the belief is not an estimate of the base rate, as one can be fully confident in a weak bias, but its evolution was more rapid on average when the base rate was more informative (Figure 2D, blue curves). We will attempt to explain the evolution of this bias by developing a theory of the two-way interaction between bias and choice—that is, the effect of bias on each decision and the effect of each decision on the estimate of the base rate of the block.

### Hierarchical Bayesian Model

**Figure 3:**
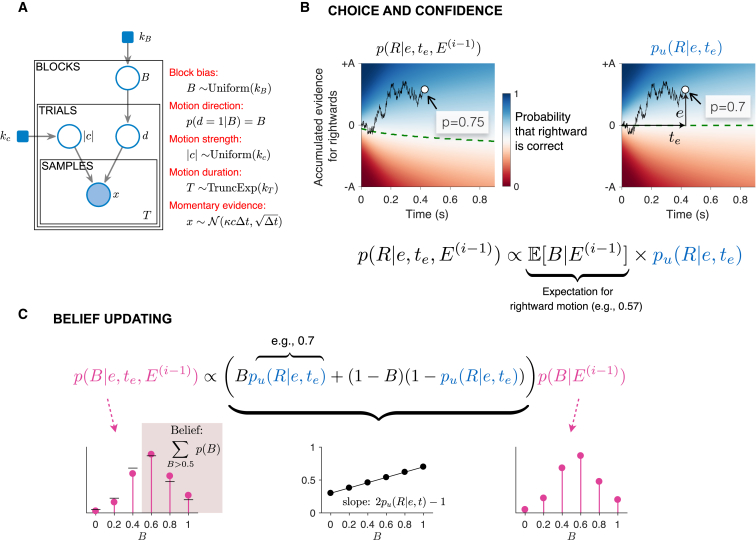
Bayesian Model (A) Graphical representation of the hierarchy of causes that give rise to a sample of momentary motion evidence. The shaded areas are observed variables, the unshaded areas are unobserved variables, and the filled squares are fixed hyperparameters. The block bias is sampled from a uniform distribution over six possible values (parameterized by $k_{B}$). B is the block’s base rate, which sets the prior probability over motion direction (*d*) for the entire block. The motion strength (|c|) is sampled on each trial from a uniform distribution over six possible values (parameterized by $k_{c}$), independent of the block’s base rate. In the experiment, |c| and *d* set the probability that a dot plotted at time *t* will be replotted at time *t* + 40 ms toward the right or left target. The duration of motion (*T*) was sampled from a truncated exponential distribution. In the model, it is assumed that the momentary evidence follows a Gaussian distribution with a fixed variance (Δt) and a mean that depends on |c| and *d*. (B) Bounded evidence-accumulation model of choice and confidence. The decision is made when the accumulation of momentary evidence (*e*) reaches a bound (±A) or the stimulus is curtailed, as in the sample trace. The two color maps show the probability that rightward would be the correct choice based on accumulated evidence *e* at time $t_{e}$, under different assumptions about the base rate: (left) expectation of the base rate is 0.57 in favor of rightward; (right) based rate is unbiased (0.5). The dashed green line marks the decision boundary (p(R)=0.5). We refer to the right map $\left(p_{u}\left(R|e,t_{e}\right)\right)$, as the counterfactual posterior probability of rightward. The left map is formed by multiplying the counterfactual posterior by the expectation of the base rate, given the evidence from the previous trials $\left(E^{\left(i-1\right)}\right)$. The normalization constant assures that the posterior over direction sums to unity. (C) Belief updating. To update p(B), we multiplied the distribution over base rate from the previous trial (shown on the right) by the expression inside the parenthesis. This expression is a linear function of *B*, with a slope given by the counterfactual posterior (center plot). The left panel shows the updated distribution after the multiplication and normalization. For reference, the horizontal black lines indicate the distribution over base rates from the previous trial. The normalization constant ensures that p(B) sums to unity. The belief that the block is biased in favor of rightward is given by the sum of p(B) for B>0.5 (shading).

We developed a hierarchical Bayesian model in which subjects maintain a probability distribution over the base rate, p(B), within a block and use this knowledge to influence both their choice and their confidence within a trial. As we will see, the optimal way to update p(B) is to use a counterfactual form of confidence—the probability that a choice rendered on the evidence would be correct if the base rate were unbiased (i.e., B=0.5). We develop this idea in Figure 3 and provide a mathematical derivation in the STAR Methods.

Figure 3A shows the hierarchy of causes that give rise to a sample of motion evidence, *x*. All trials are affected by the base rate, *B*, assigned to the block, and *B* itself is sampled from a uniform distribution. The bias in the block establishes the prior probability of direction of motion (d=sign(c), i.e., R or L) on each trial. The strength of motion (|c|) and the stimulus duration (*T*) are determined probabilistically for all trials (independent of block). The direction and strength of motion specify the stationary stochastic process comprising samples of evidence, *x*. We assume that *x* is a sample from a Gaussian distribution with mean = κcΔt and variance equal to the sample period (Δt). The parameter κ reflects the signal to noise of the evidence samples. To make a decision, the brain accumulates samples until either the accumulated evidence (*e*) reaches a threshold at ±A or the motion stimulus ends (Figure 3B). The time of this last sample is denoted $t_{e}$. If leftward and rightward motion are equally likely, the decision about direction should be determined by the sign of *e*, and the probability that the motion is rightward is determined by *e* and $t_{e}$ (Kiani and Shadlen, 2009). The heatmap (Figure 3B, right) shows the probability that the direction is rightward for all possible combinations of *e* and $t_{e}$: $p_{u}\left(R|e,t_{e}\right)$. The example trial (open circle) would have led to a rightward choice with probability 0.7 of being correct. The top half of the map thus provides a lookup table for confidence in a rightward choice. Leftward choices would arise when the evidence (*e*) is less than 0, and the probability that this choice is correct is 1 minus the values displayed—that is, the top half of the map reflected vertically across the horizontal green line. These statements about direction choice, probability of left/right and confidence apply only if leftward and rightward motion are equally probable—hence, the subscript, *u* (for unbiased), in $p_{u}\left(R|e,t_{e}\right)$. This condition does not occur in our experiment, but the mapping plays a role. We refer to the mapping on the right as a counterfactual posterior or counterfactual confidence.

In the experiment, one direction within a block is always more likely than the other, and this affects the probability that the direction is right (or left) given $\left\{e,t_{e}\right\}$. For example, if the base rate, *B*, favors rightward, it might give rise to the map in Figure 3B (left). Notice that the decision criterion (dashed green line) dips to negative values of *e*. The mapping to confidence is altered as well. The same amount of evidence leading to a right choice (circle) now corresponds to 0.75 probability of being correct. The calculation supporting this map factors neatly into the expectation of the base rate multiplied by the counterfactual posterior—the probability that the direction would be rightward if the two directions were equally likely. The expectation of the base rate is informed by knowledge obtained on the previous trials in the block, represented by $E^{\left(i-1\right)}\equiv {\left\{e,t_{e}\right\}}_{1\text{..}i-1}$. In the example, the expectation of *B* is 0.57. The confidence is 0.57×0.7 divided by the sum of this term plus (1−0.57)×(1−0.7), where the last product is $p\left(d=L|E^{\left(i-1\right)}\right)p_{u}\left(L|e,t_{e}\right)$. The arithmetic yields approximately 0.75, which is also the posterior probability of right. Put simply, on the current trial, use the evidence, deliberation time, and the estimate of the base rate (from previous trials) to make the decision and assign its confidence.

The remaining question is how knowledge of the base rate is updated. According to the hierarchical Bayesian model, the subject begins the block with a prior over the six possible base rates, $p_{0}\left(B\right)$. The true prior is uniform, but we allow for the possibility that subjects do not internalize this correctly. Figure 3C shows how these values are updated. As the graphical model (Figure 3A) makes clear, inference about *B* is arbitrated solely by the direction of motion, *d*. Key to the update is that the subject should not use the estimate of direction that they report but the probability of each direction under a neutral prior (B=0.5), in other words, the counterfactual posterior. This yields the update rule illustrated in Figure 3C, which depends on two components that change across trials: the posterior for rightward (and its complement for leftward) and the current estimate of the distribution of *B*. The use of the posterior can be visualized as a line (Figure 3C, center) that is then point-wise multiplied by the current p(B) (Figure 3C, right). The six probabilities are scaled to sum to unity (Figure 3C, left). Note that the updated distribution after one trial becomes the initial distribution for the next one (i.e., $p\left(B|E^{\left(i\right)}\right)\equiv p\left(B|e,t_{e},E^{\left(i-1\right)}\right)$). Again, the full derivation of the expressions is in the STAR Methods.

To appreciate why the Bayesian solution uses the counterfactual posterior to update p(B), consider the following example. Suppose that the participant has acquired a slight bias for rightward and is then presented with a sequence of stimuli of 0% coherence. The participant will tend to report rightward more often because the bias exerts a stronger influence when the evidence is weak. Therefore, if true posterior (confidence) is used to update the bias, the bias would tend to increase until the decision maker is certain that the block contains a rightward bias, even though the sensory evidence is ambiguous. As formalized by the expression in Figure 3C (and in the STAR Methods, Equations 6, 7, 8, and 9), the correct approach is to update the belief based on the likelihood that the evidence $\left(e,t_{e}\right)$ had been obtained in a trial with right or left motion or, equivalently, the confidence that the subject would have under a neutral (i.e., uninformative) prior. In short, the counterfactual posterior circumvents the problem of a self-reinforcing prior, what might be thought of as double counting.

This completes both parts of the theory: (1) how to incorporate one’s estimate of bias into the choice and confidence about direction and (2) how to update the estimate of bias (including the confidence in this estimate) based on this experience.

### Fits of the Bayesian Model to Choice and Confidence

Our main hypothesis is that participants modify their bias according to the counterfactual confidence they have in their decision. To test this hypothesis, we use the Bayesian model described in the previous section to fit each subject’s choices and confidence reports. We compared this model to two alternative models, one in which participants update their bias based only on the frequency of left and right choices (i.e., ignoring confidence) and another in which participants used confidence (instead of its counterfactual) to update the bias. After comparing the goodness of fit of the three models for the motion direction reports, we use the best-fitting model to predict—without additional degrees of freedom—the explicit reports of the belief that were not used in the model fitting.

**Figure 4:**
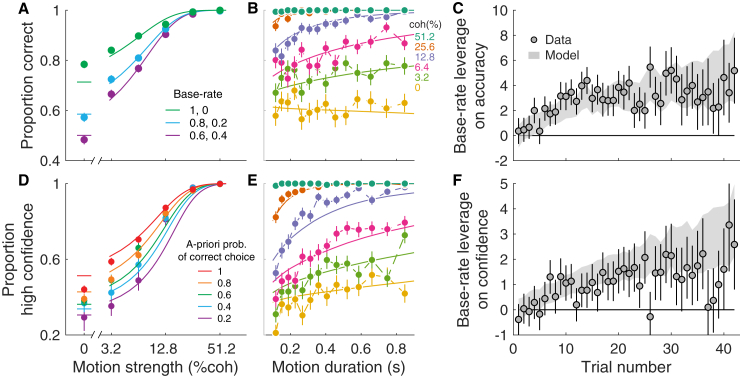
Fits of the Bayesian Model to Choice and Confidence (A) Proportion of correct responses as a function of motion strength, split by the base rate of the block (same data as Figure 2B). Solid lines are model fits. (B) Choice accuracy as a function of duration of the motion stimulus, split by motion strength. Symbols are mean ± SE, and solid lines are model fits. Points are quantiles (∼157 trials per point). (C) The influence of the base rate on accuracy increased with trial number within blocks. Symbols are the fitted coefficients (±SE) from logistic regression fits to the data ($\beta_{6}$, Equation 21). The shaded area is the SE estimated from the model (see STAR Methods). (D) Proportion of high-confidence responses on correct trials as a function of motion and base rate. The colors represent different levels of bias relative to the chosen direction (similar to Figure 2B). Symbols are mean ± SE across trials. Solid lines are model fits. (Figure S3 shows the error trials.) (E) Proportion of high-confidence responses as a function of the duration of the motion stimulus. Correct trials only. Same grouping of trials as in (B). (F) The influence of the base rate on proportion of high-confidence responses increased with trial number within blocks. Symbols are the fitted coefficients (±SE) from logistic regression fits to the data ($\beta_{9}$, Equation 22). The shaded area is the SE estimated from the model (see STAR Methods). See Figure S3 for single-participant data.

We used the sequence of stimuli (motion coherence and duration) on each trial to maximize the likelihood of the choice (left or right) and confidence (high or low) on each trial (see STAR Methods). The model constructs a hidden “latent” representation of the subject’s knowledge of the base rate, p(B), as it evolves with each trial. As shown in Figure 4A, subjects’ choice accuracy was explained by the Bayesian model. The points, which are identical to those in Figure 2B, combine data over the entire block, for both directions, and for all stimulus durations. The more informative the base rate, the more it can be relied upon to improve accuracy. The model quantitatively explains the degree to which the base rate was learned and incorporated by the participants (see Figure S3 for individual subjects). Figure 4B shows the same accuracy data but as a function of viewing duration for each motion strength. Here the data combine all base rates. Note that accuracy improves as a function of viewing duration for all informative (>0%) coherences (Equation 21; $p<10^{-8}$, t test, $H_{0}:\beta_{2}=0$). This improvement is consistent with bounded accumulation of noisy evidence, where the bound curtails improvement at longer viewing durations, consistent with previous studies (Kiani et al., 2008, Zylberberg et al., 2012). These two graphs are informative cross sections of a rich dataset.

Knowledge of the base rate, *B*, was acquired gradually during each block of trials. Figure 4C demonstrates the time course of the changes in choice accuracy. We used logistic regression to estimate the leverage of base rate on accuracy for each trial in a block (STAR Methods, Equation 21). The ordinate shows the leverage of *B* on accuracy after accounting for motion strength. It supports the model-free assertion that bias-dependent accuracy is learned over the course of the block (Equation 21; $p<10^{-8}$, likelihood ratio test, $H_{0}$: all $\beta_{6}=0$), and it shows that this rate is consistent with expectations of the Bayesian model (gray shading).

The model was also fit to explain the subject’s confidence in the direction report. For each subject, we tried to explain the probability that their confidence was high or low, relative to a criterion setting (see STAR Methods). The model produces an estimate of confidence that depends on the stimulus (strength and duration), the subject’s choice, and the model’s current estimate of the base rate (Figure 3C). It did not incorporate the subject’s belief reports. The lower row of Figure 4 shows the confidence fits in a way that parallels the accuracy analyses. The trial groupings are the same as in the corresponding accuracy plots (upper row), with one exception. The block base rate (Figure 4D) are expressed as the *a priori* probability that the direction of motion supports the choice that the participant made (correct choices only), as in Figure 2C. The model captures the important features of the data. Larger *a priori* probability of a correct choice increased confidence, and this effect was more apparent at the weaker motion strengths (Figure 4D). Confidence also varies as a function of stimulus motion strength and duration (Figure 4E). There is some mismatch with the model at 12.8% coherence (Figure 4D), especially for the 300–600 ms durations (Figure 4E). The effects build up gradually as a function of trial number in the block (Figure 4F), showing that this rate is consistent with expectations of the Bayesian model (gray shading).

We compared the Bayesian model against two alternatives, which differ in the way knowledge of the base rates is updated across trials. As indicated in Figure 3C, a Bayesian observer will update her knowledge about the base rate after each trial using the expression,

Equation 1 $$p\left(B|e,t_{e},E^{\left(i-1\right)}\right)\propto \left(B\nu_{r}+\left(1-B\right)\nu_{l}\right)p\left(B|E^{\left(i-1\right)}\right)$$

where $\nu_{r}=1-\nu_{l}$ is equal to the counterfactual posterior for rightward motion, $p_{u}\left(R|e,t_{e}\right)$. In the first alternative, the participant updates p(B) based on the frequency of left and right choices, weighting all choices equally (i.e., ignoring confidence). This is implemented by making the evidence $\nu_{r}$ equal to 1 for right choices and 0 for left. We refer to this model as the Choice-only model. In the second alternative model, the evidence for updating the bias is the confidence that the participant reports in each trial rather than the counterfactual confidence that would have been reported under a neutral prior. To model this, we made $\nu_{r}$ equal to the confidence that the subjects report when they chose rightward, thus equal to $p\left(R|e,t_{e},E^{\left(i-1\right)}\right)$, and to one minus confidence when subjects chose left. We refer to this model as the Choice-confidence model. As mentioned earlier, this model is suboptimal because the evidence for updating the bias is corrupted by the bias itself.

A model comparison showed that the Bayesian model is the one that best fit the data for all participants (combining across participants, the difference in log likelihood was 70 and 75 for the Choice-only and Choice-confidence models relative to the Bayesian model; see Table S1 for data from individual participants). The three models have the same number of parameters (Table S2), and thus, the same result is obtained with measures that penalize the goodness of fit by the number of free parameters (e.g., the BIC or AIC). The model comparison supports the interpretation that participants used a graded measure of certainty in the decision to update their beliefs about block bias. The support for the Bayesian model is particularly strong when we combine the likelihoods over the different participants. Note that the support for the Bayesian model derives from a model comparison that uses only the choices and the confidence in the motion direction decision.

### The Evidence for Belief Updating: An Empirical Approach

**Figure 5:**
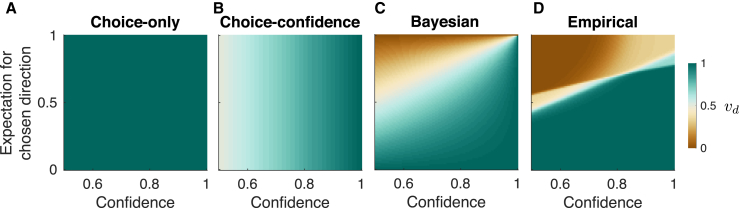
Descriptive Rendering of the Rule to Update Knowledge of the Base Rate The Bayesian model and its alternatives can be characterized by the way they update p(B) based on the choice-confidence and the expectation of the base rate, expressed relative to the choice taken ($E\left[B_{d}\right]\equiv p\left[d|E^{\left(i-1\right)}\right]$, similar to *a priori* probability correct). The strength of the update is parameterized by $\nu_{d}$. (A) For the Choice-only model, $\nu_{d}$ is always equal to 1. (B) For the Choice-confidence model, $\nu_{d}$ only depends on the reported confidence. (C) For the Bayesian model, $\nu_{d}$ is the counterfactual confidence, which is a function of both reported confidence and $p\left(d|E^{\left(i-1\right)}\right)$. (D) For the Empirical model, we used a flexible mapping (Equation 16) of confidence and $p\left(d|E^{\left(i-1\right)}\right)$ to $\nu_{d}$ that best fit the data.

The three models represent distinct alternatives for updating: Choice-only, Choice-confidence (i.e., choice weighted by confidence), and Bayesian. The model comparison, based on fits to choice and confidence, provides support for the Bayesian model, but the exercise fails to capture the qualitative differences in these models. Here we pursue a more general approach (Figure 5). The critical issue differentiating these models is the way they update the knowledge of the base rate, p(B). In Equation 1, this is captured by the term $\nu_{d}$ that multiplies the six possible values of the base rate (where *d* stands for the chosen direction; i.e., $\nu_{d}=\nu_{r}$ or $\nu_{l}$). We can view each of the three alternatives as different instantiations of $\nu_{d}$, which depends on two quantities: (1) the confidence that choice is correct, $p\left(d|e,t_{e},E^{\left(i-1\right)}\right)$, and (2) the expectation about the direction of motion before seeing the stimulus presented on the trial, $p\left(d|E^{\left(i-1\right)}\right)$. These two quantities are the abscissa and ordinate of the graphs in Figure 5. The color depicts the updating term $\nu_{d}$ as specified by the three models (Figures 5A–5C) or fit to the data using an arbitrary combination of the two terms, which we refer to as an “Empirical model” (Figure 5D). In the Choice-only model (Figure 5A), $\nu_{d}$ is a constant, implying that the update depends only on the choice. In the Choice-confidence model (Figure 5B), $\nu_{d}$ depends only on the confidence. In the Bayesian model (Figure 5C), $\nu_{d}$ is the counterfactual confidence, which manifests as an interaction between confidence and the expectation of the base rate. Intuitively, this is because the confidence is an expression of the posterior probability, whose relationship to the counterfactual posterior requires removal of the prior—that is, the expectation of the base rate. We next derive an empirical mapping that best accounts for the data.

To produce Figure 5D, we used a flexible five-parameter mapping (Equation 16) to relate $\nu_{d}$ to the two quantities represented on the abscissa and ordinate. Unlike Figures 5A–5C, in which $\nu_{d}$ is derived from theory, in Figure 5D, $\nu_{d}$ is obtained by fitting the data, as in Figure 4, by maximizing the likelihood of the choice and confidence data. The key difference is now we incorporate additional terms to specify $\nu_{d}$ (see STAR Methods). The resulting empirical map (Figure 5D) resembles the Bayesian map in Figure 5C. For a fixed level of confidence, knowledge of the base rate is affected more strongly when the prior expectation of the decision direction is less supportive of the choice (vertical gradient). For example, when this expectation opposes the chosen direction (i.e., lower half of the map), the update is stronger. Intuitively, this is because the reported confidence is a function of both the stimulus strength and the prior knowledge. Therefore, if the prior opposes the choice (expectation <0.5), then the confidence that the subject would have under a neutral prior (expectation=0.5) must be greater than the one reported. The exercise complements the conclusions drawn from model comparison by adopting a more open-ended answer to the question of what combination of confidence and prior expectation best supports the updating of p(B) consistent with choice. The answer is that confidence is not sufficient but is adjusted by prior expectation in a way that emulates the conversion to counterfactual confidence.

We also explored the possibility that our participants used a combination of the Bayesian and Choice-confidence models to update their knowledge about the base rate. To test this alternative, we fit a new model, similar to the Empirical model presented in Figure 5 but in which $\nu_{r}$ was set equal to $\beta p_{u}\left(R|e,t_{e}\right)+\left(1-\beta \right)p\left(R|e,t_{e},E^{\left(i-1\right)}\right)$. If β is one, the model is identical to the Bayesian model, and if β is zero, it is identical to the Choice-confidence model. For intermediate values of β, the model updates its knowledge of the base rate using a combination of the values of $\nu_{r}$ used by both models. We fit the model to data just as for the Bayesian and Choice-confidence models, but with β as an additional free parameter. For every participant, we found that β was greater than 0.9994. This provides additional support for the Bayesian model over the Choice-confidence model (and over a combination of the two). Having provided further support for the Bayesian model (counterfactual confidence), we now use this model to predict the subjects’ belief reports.

### Belief Predictions from Fits to Choice and Confidence

To establish fits of the Bayesian model on the basis of each subject’s choices and confidence, we relied on an estimate of the base rate (E[B], Figure 3B) derived from the distribution, p(B). This distribution was updated in accordance with Bayesian theory using the previous decision and counterfactual confidence. Although subjects reported their belief that the block was biased to the right or left, we did not use these reports to fit the model. Thus, we can now ask how well the model predicted these reports. According to the model, the belief that the base rate favors rightward ought to correspond to the sum of p(B) for values of *B* greater than 0.5 (Figure 3C),

Equation 2 $$\text{belief}\equiv \sum_{B>0.5}p\left(B\right)$$

**Figure 6:**
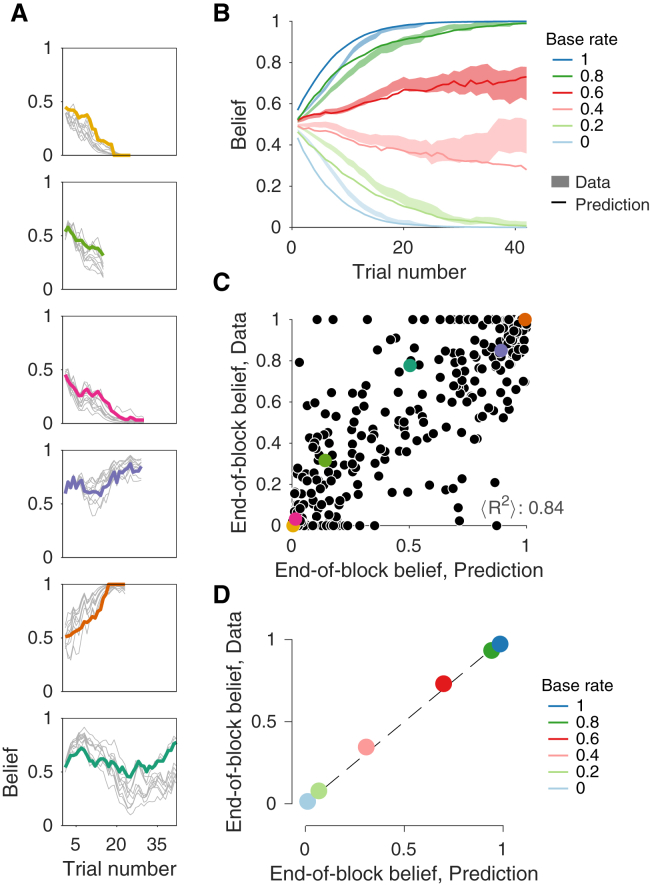
The Bayesian Model Predicts Belief Reports (A) Evolution of beliefs for six example blocks. The colored thick lines are the data. The thin lines represent predictions from ten simulations of the Bayesian model using the same sequence of trials (motion strength, duration, and direction) as seen by the subjects. They differ because the evidence is sampled randomly on each trial $\left(\left\{e,t_{e}\right\}\right)$, which may lead to differences in choice and confidence. (B) Evolution of belief as a function of the trial number within the block. Lines are predictions of the model, and the shaded areas show the SE of the data (as in Figure 2D). (C) Predicted belief for the last trial of each block plotted against the data from the same block. The colored circles identify the six blocks shown in (A). The predictions (abcissa) are from one simulation of the full experiment; the average R^2^ indicated in the panel is an average over 200 simulations. (D) Same comparison as in (C) but averaged by actual base rate. Error bars are SE (usually smaller than the data points); dashed line is identity. See also Figures S4 and S5.

Figure 6A shows the predictions and data for the belief that the block has a rightward bias for a few individual blocks. Each panel shows data from one randomly chosen block (colored trace). The gray lines show the belief produced from simulations of the block, using the same stimuli in the same order of the block. The simulated trials lead to different states of the accumulated noisy evidence, hence different choices and confidence, which in turn affect the evolution of p(B). Each gray line shows the evolution of belief for a different run of the model. For both the model and the participants, the belief after the first decision is close to ½. As more decisions are made, the belief tends to become more certain.

This is seen more clearly when we average the belief for the six different levels of base rate (Figure 2D), redrawn here as colored areas showing the SEM across trials (Figure 6B). The thin lines in Figure 6B are the predicted time course from the Bayesian model. Subjects’ belief reports initially lagged the predictions, but by trial 20, the data and predictions are almost perfectly aligned. This agreement was evident across the individual blocks. To quantify this, we compared the reported belief on the last trial of each block to the predicted belief on these trials (e.g., the average of the termination of the model simulation in Figure 6A). The scatterplot (Figure 6C) displays this comparison for all 485 blocks (the examples in Figure 6A are identified by color). Moreover, the average belief on the last trial of each block was almost identical to the predictions of the Bayesian model when grouped by base rate (Figure 6D). The agreement between data and predictions is remarkable considering that the predictions were based only on the fits of the model to the direction choice and confidence.

**Figure 7:**
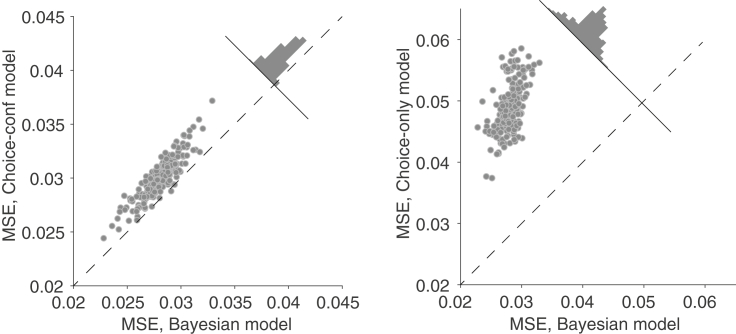
Comparison of Model Predictions of Belief at End of Block For 200 simulations of the full experiment for each of the three models, we predicted the belief at the end of each block and computed the mean squared error between the predictions and the data. Each point represents a pair of models that were simulated with the same sequence of stimuli: (left) Bayesian model versus Choice-confidence model; (right) Bayesian model versus Choice-only model. The histogram shows the distribution of the difference in MSE across the 200 simulations. The prediction errors were consistently smaller for the Bayesian model.

While the belief predictions on Figure 6 were produced by the Bayesian model, similar predictions were made by the Choice-confidence model and even by the Choice-only model (Figure S4). This implies that the agreement between model and data in Figure 6 cannot be taken as support for the Bayesian model. To compare the accuracy of the predictions made by the different models, each model was simulated 200 times on the full experiment, and, for each simulation, we compute the mean squared error (MSE) between model and data using the belief at the end of each block. Figure 7 shows the MSE comparisons between the Bayesian and the other two models. Each point represents a pair of simulations using the identical sequence of motion stimuli. The beliefs predicted by the Bayesian model were consistently better than those of the Choice-confidence (Figure 7, left; $p<10^{-8}$, paired t test) and the Choice-only (Figure 7, right; $p<10^{-8}$, paired t test) models, providing additional support for the Bayesian model. We also corroborated the prediction that stronger motion, associated with higher confidence on average, should lead to a larger change in the belief that the block is biased to the right or left (Figure S5). The analysis does not discriminate between the Bayesian and the Choice-confidence models, but it is inconsistent with the Choice-only model.

**Figure 8:**
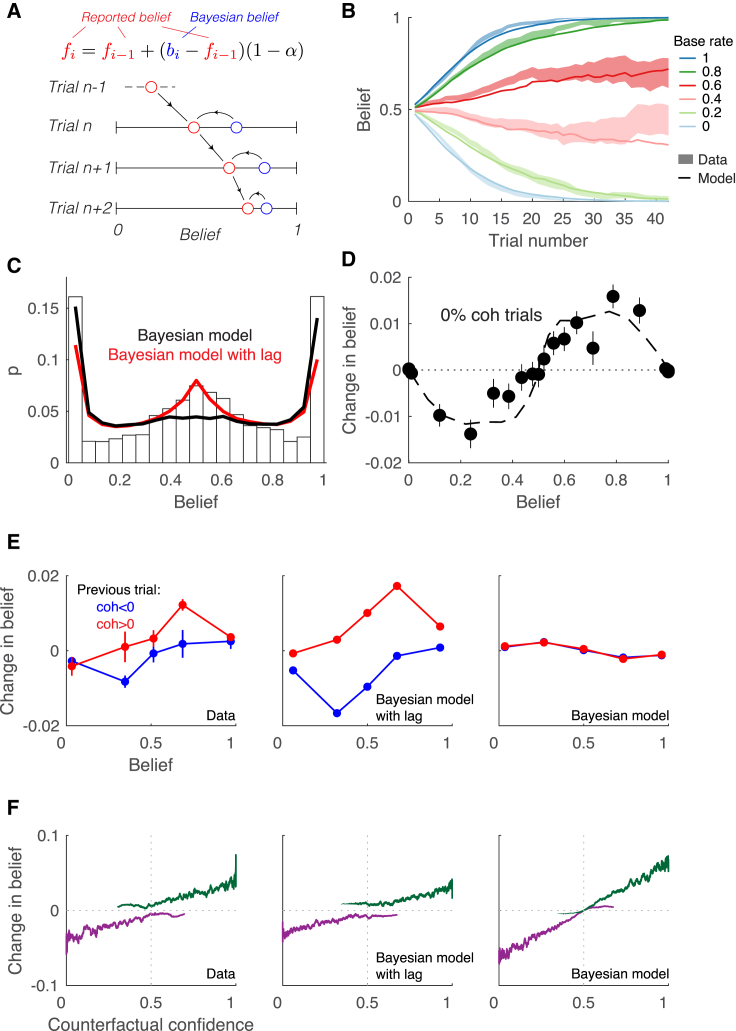
A Lag in the Belief Reports (A) Sketch of the model with lagging belief reports. $f_{i}$ is the reported belief on trial *i*, and $b_{i}$ is the belief that would have been reported under the Bayesian model. If the time constant of the belief (α) is zero, the reports are equal to those of the Bayesian model; if it is one, the belief is always ½. (B) Evolution of belief as a function of the trial number within the block. Lines are predictions of the model with lag. The shaded areas show the SE of the data. (C) Distributions of belief reports from the participants (histogram), and the Bayesian model with and without lag. The Bayesian model cannot explain the large number of trials with belief near ½, but the mismatch is reduced with the addition of the lag. (D) Change in belief from one trial to the next, as a function of the current belief. The analysis only includes trials with 0% motion strength. Data were binned into 20 percentile groups. For both model with lag (dashed line) and data (points), beliefs are, on average, updated in the direction of the previously held belief. Error bars indicate SE. (E) As (D), but splitting trials by the sign of the motion coherence on the previous trial. Data were binned into five percentile groups. The change in belief is influenced by the direction of motion of the previous trial. The effect is replicated by the model with lag, but not by the Bayesian model. Error bars indicate SE. (F) Change in belief as a function of the counterfactual confidence inferred from the model. Data are split by choice. Lines are moving averages of 100 trials. The Bayesian model predicts no change in belief if the counterfactual confidence is ½. In contrast, changes in belief were informed by choice even when the counterfactual confidence was ½ (left), an effect that is also seen in the model with lag (middle). Figure S8 shows the same analyses for the Choice-confidence model.

One clear mismatch between predicted and observed belief settings is apparent in the early trials of the block, where there is a lag in the belief reports relative to the predictions of the Bayesian model (Figures 6B and S4). We hypothesized that this lag might arise if participants communicate each change of belief gradually over several trials, thus blurring the reports. To evaluate this proposal, we included a single additional parameter in our model, the time constant α over which the belief update affects the report (Figure 8A). This simple modification reduced the discrepancy between model and data (Figure 8B), and it explains several other aspects of the belief reports, such as their distribution (Figure 8C; incorporating the lag reduced the MSE between the reported belief and the model prediction by 2%, 28%, and 22% for the three subjects, respectively; p<0.005 for all subjects). Further, in the Bayesian model, for trials with 0% motion strength, the change of belief should be near zero on average, because counterfactual confidence is equally likely to support leftward or rightward motion. On the contrary, subjects tended to update their beliefs in the direction of the previously held biases (Figure 8D). This effect was reproduced in simulations of the model with lag. The intuition is simple: when the bias is rightward (i.e., belief > ½ ), the changes of belief from previous trials contribute to the change in belief on the current trial even when the coherence is 0%.

The carryover effects of one trial to the next can be observed more directly by splitting the change in belief by the motion strength of the previous trial, when the motion strength in the current trial is 0%. Consistent with our interpretation, the change in belief at 0% coherence is informed by the coherence of the previous trial, an effect that is inconsistent with both the Bayesian and the Choice-confidence models without lag (Figures 8E and S8). Further, the Bayesian model predicts that for trials on which the counterfactual confidence is ½, there should be a negligible change in belief regardless of the participant’s choice (Figure 8F, right). Instead, the data show an effect of choice even when the counterfactual confidence (inferred from the model) is ½ (Figure 8F, left). A similar dissociation by choice is observed in the Bayesian model with lag (Figure 8F, center). The chosen direction is informative about the bias of the block and thus about the direction of the change in belief that is carried over from previous trials. Note that our implementation of lag retains consistency with the Bayesian model because the internal estimate of p(B) is updated immediately to affect the next trial (via E[B]). It is just the position of the computer mouse used to express the belief that is smeared over a few trials. We verified this assertion by evaluating an alternative model in which the lag directly affects the update of p(B) (Equation 18) and therefore also choice and confidence. The best fit was a lag of zero for all subjects. This was true for the Choice-only and Choice-confidence models too and therefore does not affect the model comparison. These results indicate that the lag is specific to the communication of belief rather than the update of p(B) itself.

We have assumed that subjects can both report their belief that the block is biased (p(B>0.5)) and represent the underlying magnitude of the base rate (E[B]). To confirm this, we analyzed the choices on 0% coherence trials in which they were close to certain about the bias of the block (>0.95 or <0.05 on the previous trial). If the subject’s representation of base rate matches that of the block, then on these trials with little net motion, the subject should report more frequently in the direction of bias when the base rate is more informative despite the belief being equally strong. Indeed, subjects were more likely to report the direction of the belief when the base rate was more extreme (Equation 24; $p<10^{-6}$, t test, $H_{0}:\beta_{1}=0$). This analysis presents model-free evidence that participants represent not only the quantity that they are asked to report (belief) but also a measure of the magnitude of the base rate.

## Discussion

Base rate, prevalence, and prior probability distribution are examples of statistical regularities that ought to shape our decisions. A decision maker must learn these regularities from sources, such as census or epidemiological research, or through learning. We have shown that decision makers can infer such regularities from their own decisions and use the evolving knowledge of the base rate—its prior probability distribution—to make better decisions, even during the learning process itself. Remarkably, they did this without feedback about the validity of their decisions, which were, for the most part, difficult. Indeed, more than half of the decisions were about weak motion that would, under neutral priors, give rise to correct choices on less than 75% of trials—that is, just better than guessing. Such difficult trials are the very ones that benefit the most from knowledge of the base rate. We showed that subjects incorporated an evolving estimate of base rate into these decisions and their confidence while at the same time exploiting the decision to update knowledge of the base rate.

We observed that the influence of the base rate on our three behavioral measures—decision, confidence, and belief—developed gradually during the block. This was evident in the two ways by which we evaluated the learning of the base rates: implicitly, through their influence on decision and confidence, and explicitly, through the belief report—the confidence that the base rate favors rightward. Both methods gave consistent results. Indeed, we were able to fit a model to the decision and confidence reports and predict how the belief evolved during the block. This suggests that the influence of bias on the implicit and explicit reports is mediated by a common estimate, which in our model is represented by a probability distribution of possible base rates that is updated from one decision to the next. It also shows that people have introspective access to quantities that are used in the computations of a hierarchical probabilistic model, and therefore, subjective reports can be used to constrain models of decision making (Kang et al., 2017).

A theoretical analysis showed that the evidence for updating knowledge about the base rate is a “counterfactual” form of confidence, understood as the confidence that the subjects would have if left and right were equally likely (i.e., a neutral prior) and not the confidence that the subjects report. The intuition for this distinction is simple: the evidence used to modify an hypothesis should not be altered by the decision maker’s previous belief about the veracity of the hypothesis. We compared the optimal solution to two suboptimal alternatives that differ in the information used to update belief: choice-only and choice-confidence. These alternatives achieve explanations of subjects’ evolving belief that differ only subtly from the Bayesian counterfactual confidence solution, at least by eye (Figure S4, but see Figure 7), but a formal model comparison supports the conclusion that subjects used counterfactual confidence to update their bias. This conclusion was confirmed with a separate model in which we directly search over the space of transformations that map confidence and bias strength on the evidence that is used to update their knowledge of the base rate (Figure 5). The use of a counterfactual confidence for updating the bias implies the existence of different probabilistic quantities associated with the same state of accumulated evidence, a distinction that may be related to the one between confidence and visibility (Rausch and Zehetleitner, 2016).

The concept of a counterfactual posterior, and the related counterfactual confidence, demands some justification. Mathematically, the counterfactual posterior is simply the likelihood. Just because we factor the update rule to highlight this quantity does not automatically imply that the brain uses it. We believe that such a representation is neurophysiologically plausible because likelihoods are known to be represented in the brain (Yang and Shadlen, 2007, Kira et al., 2015) and can arise from simple sum and differences of neural firing rates (Gold and Shadlen, 2001, Shadlen et al., 2006, Ma et al., 2006). Moreover, it is obvious that a representation of counterfactual confidence is essential in many types of real-world decisions. For example, if instead of manipulating the prior probability of right and left, we had altered the reward such that all correct rightward choices were rewarded more than leftward, it would induce a rightward bias (Ulehla, 1966, Rorie et al., 2010). In this circumstance, it would be sensible to express both a confidence that we made the better choice and a confidence that the motion was to the right. For example, when confronted with weak motion, we would be confident that a rightward choice was best while simultaneously less confident that the net direction was to the right. The dual representation of confidence is intuitive when value induces a bias but less so when prior probability (base rate) does so, because the prior is about the motion itself. What we show here is that such a dual representation of confidence in our decision and counterfactual confidence allows us to both respond as accurately as possible and learn about the statistical regularities of the environment.

Our Bayesian model incorporates at least one element of suboptimality. Although subjects were informed that the distribution over base rates was discrete and uniform, we allowed for the possibility that the prior assumed by the subjects was not veridical. We approximated this initial prior, $p_{0}\left(B\right)$, with two parameters, which were estimated in the fits to the direction choice and confidence (see STAR Methods). All three subjects appear to have assigned a greater initial probability to base rates away from the extremes (Figure S6). This might imply that subjects were unable to override a predisposition that sequences of random samples tend to be only weakly biased, on average—an arguably sensible prior over prior probability distributions—despite direct verbal instruction. The deviation from optimality was only marginally detrimental to performance. For example, the proportion of correct responses was 85.01% in the data and 84.93% in simulations of the Bayesian model, whereas we estimate that the uniform prior would yield 87.43% correct. It could be argued that the lag in the belief reports also represents a suboptimal behavior. This would certainly be the case if the lag affected actual belief (i.e., p(B)), but that was not the case. Moreover, since participants only received feedback about the true base rate at the end of the block, there was no cost associated with delaying the report, and at the end of the block, the belief was in agreement with theory.

The lag does have an unfortunate ramification, as it limits our ability to test some qualitative predictions of the Bayesian model. For example, we would predict that the change in belief on trials with 0% coherence should average to zero regardless of the currently held belief. However, such an analysis is not possible because of the lag: the change in belief communicated by the subject was affected by changes in belief from previous trials. It would be desirable to eliminate this lag in future investigations. Preliminary investigations lead us to believe that this might be achieved using a different method to report belief using movements for which lagging does not reduce motor cost (e.g., eye movements).

Our task represents an attempt to extend our understanding of the neurobiology of simple decisions to tasks that have hierarchical structure. One tempting idea is that the same process of evidence accumulation that allows one to make a simple perceptual decision extends to higher levels and longer timescales (Kim et al., 2017, Purcell and Kiani, 2016, Glaze et al., 2015, Braun et al., 2018). In the abstract, this is true because both are effectively Bayesian, but this insight deserves further scrutiny. The idea that the brain updates p(B) in the same way that it updates a decision variable implies the accumulation of a scalar quantity across multiple trials. This strategy would furnish an optimal estimate of the base rate if the decision maker were to receive unambiguous feedback about the true direction on each trial (Laplace, 1814). However, when the decisions vary in difficulty (and thus in confidence), there is no scalar quantity that the subjects can accumulate from one trial to the next that preserves all information about p(B). To provide an intuition, suppose the subject used counterfactual confidence, signed by choice, to update p(B), and consider two scenarios. (1) A decision maker chooses rightward on the first trial and leftward on the second, both decisions made with full confidence. (2) The same choices ensue but with very low levels of confidence. In both scenarios, the running tally would be zero, but in the first, the uncertainty should be reduced more than in the second. This is because in the first scenario, it is known that the block has both right and left stimuli and therefore extreme biases are unlikely. This knowledge is captured by the normative propagation of p(B) but not by any scalar accumulation strategy. Of course, the brain might approximate p(B) using a few parameters, such as a Beta distribution (Ma, 2012).

In models of bounded evidence accumulation, the prior is often incorporated as a static shift in the starting point of the accumulation (e.g., Carpenter and Williams, 1995, Mulder et al., 2012). In our task, however, this would be suboptimal, because as time elapses, the same level of accumulated evidence is a less reliable marker of decision accuracy, and thus the bias should be dynamic (Hanks et al., 2011, Huang et al., 2012, Drugowitsch et al., 2012). Indeed, Hanks et al. showed that monkeys and humans are sensitive to the time dependence of the influence of the prior when making decisions similar to ours. In their implementation, a dynamic bias signal gradually displaces the DV toward a decision bound (the one representing the most likely choice) and away from the other. This approximation was motivated by observations in the neural recordings and reaction times. Here we pursued a normative approach and thus replaced this approximation with a representation of likelihood and its mapping, induced by the prior, to the dynamic posterior probability that motion is to the right or left (e.g., Figure 3B).

The present study complements several recent studies which have addressed the manner in which a decision maker can sense a change in the statistical structure of the environment (Summerfield et al., 2011, Behrens et al., 2007, Norton et al., 2017, Nassar et al., 2010, Yu and Cohen, 2008, Anderson and Carpenter, 2006), similar to the change in base rate that occurs (with probability 5/6) at the beginning of each block in our experiment. Whereas we provided explicit instruction that the block changed and no feedback about the decisions made during the block, these complementary studies provide no explicit feedback that the environment has changed. Instead, they provide feedback about the success or failure of the decisions made in the new environment (cf. Kim et al., 2017). The common theme is that statistical inference occurs over two temporal scales, one concerning individual decisions and the other the statistical regularities about the environment in which these decisions are undertaken. The key contribution of the present study is to show that counterfactual confidence, derived from the decision at hand, informs the evolving knowledge of statistical regularities over the longer timescale of many decisions. In the tomato problem introduced earlier, the sorter would apply whatever knowledge of the base rate she has to each tomato. It would tend to influence the borderline cases most. With each decision, knowledge of the proportion of ripe and unripe would be updated using confidence in the tomato itself, stripped as it were, from the influence of base rate, as if imagining a box with equal proportions of ripe and unripe fruit. The sorter might use this evolving knowledge to tell a co-worker that a crate seems to contain mostly unripe fruit and the degree to which she believes this to be true. She could also report her estimate of the proportion itself, but as in our experiment, that will also be evident in the decision about the next sample and the confidence that it has been sorted correctly.

Without immediate feedback from the world, confidence is all we know about the veridicality of our assertions. Recent progress in understanding the mechanisms for deciding and assigning a degree of confidence to isolated decisions has set the stage to study the role of confidence in more complex tasks, especially those that involve multiple steps (Gold and Stocker, 2017, Zylberberg et al., 2011). It has been suggested that confidence plays a key role in assigning blame to different sources of evidence after an error (Purcell and Kiani, 2016, McGuire et al., 2014), controlling how much effort and time to invest in a decision that depends on the success of a previous decision (van den Berg et al., 2016), combining decisions in a hierarchy to maximize reward (Lorteije et al., 2015), and guiding perceptual learning in the absence of feedback (Guggenmos et al., 2016). Confidence may also play a role in combining individual opinions with those of a group according to their reliabilities (De Martino et al., 2017, Park et al., 2017, Bahrami et al., 2010). Our work adds to these studies by showing that confidence mediates the bidirectional process by which decisions both inform and are informed by a dynamic estimate of a base rate. It does so by drawing implicitly on counterfactual knowledge about confidence associated with a setting that is inapplicable to the decision at hand. Counterfactual reasoning is thought to play a role in slow, deliberative decision making, where it relies on narrative devices or playing through imagined scenarios. It is intriguing to consider that a more automatic form of counterfactual reasoning, such as we have demonstrated here, arises in a “thinking fast” (Kahneman, 2011) mode, where it conforms to normative Bayesian principles.

## STAR★Methods

### Key Resources Table

**Table:** 

REAGENT or RESOURCES	SOURCE	IDENTIFIER
**Deposited Data**		

Behavioral data	This paper	https://dx.doi.org/10.17632/vzhs4m5573.1

Software and algorithms		

MATLAB	MathWorks	R2017b
Custom code	This paper	https://github.com/arielzylberberg

### Contact for Reagent and Resource Sharing

Further information and requests for resources should be directed to and will be fulfilled by the Lead Contact, Ariel Zylberberg (ariel.zylberberg@gmail.com).

### Experimental Model and Subject Details

Three human participants (2 male, 1 female; age 24-35) gave informed consent and participated in the experiment. The experimental protocol was approved by the local ethics committee (Institutional Review Board of Columbia University Medical Center). All participants had normal or corrected-to-normal vision, and were naive about the purpose of the study. Owing to sample size, no gender specific analyses were performed.

### Method Details

#### Behavioral Task

Stimuli were displayed on a CRT monitor (ViewSonic, PF790) with a refresh rate of 75 Hz. A head and chin rest ensured that the distance between the participants eyes and the monitor’s screen was 48 cm. We tracked the position of the eye with an Eyelink 1000 eye-tracker.

Subjects performed a motion direction discrimination task in blocks of trials. They were given no information about their performance until the end of each block. The number of trials in each block was sampled from a truncated geometric distribution (p = 1/15) with a minimum of 15 trials and a maximum of 42 trials. Within each block of trials, one of the motion directions (leftward or rightward) was more probable than the other. The base rate of each block was sampled with equal probability from the set B∈[0,0.2,0.4,0.6,0.8,1], where *B* indicates the proportion of rightward directions. That is, given a block of $n_{tr}$ trials, the number of trials with rightward motion was $B\times n_{tr}$, rounded to the nearest integer. The order of trials was randomly permuted. Subjects were informed about the set of base rates and that they were equally probable for each block, but they were not informed about the base rate of each block until the block was completed.

Each trial started with participants fixating a small point (diameter of 0.33 deg visual angle) at the center of the screen. After 0.5 s, two target arcs (2π/3 radians) of an imaginary circle (radius 10 deg) appeared to the left and right of the fixation point (Figure 1; similar to Zylberberg et al., 2016). The Subjects had to select the left or right target to indicate leftward or rightward motion, respectively. Subjects used the upper extreme of targets to indicate full decision confidence (100% certainty that the direction choice was correct) and the lower extreme to indicate guessing (50% certainty). Intermediate values represent intermediate levels of confidence. As a visual aid, the targets were colored from green to red from top to bottom (Figure 1).

After a variable duration of 0.3 - 1 s (truncated exponential; τ = 0.5 s), the random dot motion was presented in a virtual aperture centered at fixation and subtending 5 degrees of visual angle. The dot density was 16.7 dots/deg^2^/s and the displacement of the coherently moving dots produced an apparent speed of 5 deg/s. In each video frame, a subset of the dots presented 40 ms earlier were displaced toward the right or left target, depending on the motion direction for that trial. The details of the stimulus generation process can be found in previous studies (e.g., Roitman and Shadlen, 2002). The probability that a dot moved coherently as opposed to randomly varied from trial to trial according to the motion strength (|c|), sampled uniformly from the set [0, 3.2, 6.4, 12.8, 25.6, 51.2]%. To keep the motion strength approximately balanced within each block, we constructed a list of motion coherences by repeating the six unique coherence values until the first multiple of six that is larger than $n_{tr}$; then we shuffled these motion coherences, and assign the first $n_{tr}$ values to the trials in the block. Note that the distribution of motion strengths within a block is independent of the base rate.

The random dot motion disappeared after 0.1-0.9 s (truncated exponential, τ = 0.6 s), together with the fixation point, and the participant then reported her decision with a computer mouse. Subjects were free to move the cursor until satisfied with their choice and confidence report (signaled by pressing the spacebar). As a visual aid, when the cursor approached one of the arcs, a marker was shown on the arc that was closest to the cursor position together with a number between 50 and 100, indicating the probability (as percentage). See Video S1 for the actual stimulus display.

After the choice and confidence reports about motion direction, subjects reported the degree to which they considered the block to be biased in one direction or the other, indicated on a continuous horizontal scale. The leftmost extreme indicated full certainty in a left bias (belief = 0) and the rightmost extreme indicated full certainty in a right bias (belief = 1). The in-between points represented intermediate levels of certainty, with the center of the scale indicating maximal uncertainty, meaning that subjects considered equally likely that the block was biased in one direction or the other. We used color gradients as a visual aid, with a scale that went from white to black and then back to white. Subjects reported their decision by moving a small triangle to the desired location before pressing the spacebar to record the belief as the current position of the triangle (see Video S1). For the first trial of a block, the triangle was rendered at the center. For all subsequent trials it reappeared at the location where it was set in the previous trial. Subjects were only provided with feedback after a block had been completed. Feedback comprised a single screen indicating whether the block was biased to the left or to the right, the probability that a trial was biased in this direction (60, 80 or 100%), and the proportion of trials in the block that the subject reported correctly. An example feedback screen is shown in Figure 1B.

Participants performed the task across multiple sessions (9 to 14, usually 1 session/day). They usually completed 12 blocks per session. Across days, participants completed 165, 150 and 170 blocks, respectively, for a total of 4564, 4051 and 4554 trials. Before performing the main experiment, participants were trained to discriminate the direction of random dot motion. During training, the base rate was fixed and equal to ½. Participants first performed a version of the task in which they were only required to report the motion direction (436, 432 and 936 trials per participant). During a second stage of training, they also reported their confidence, using the same numerical scale as in the main experiment (961, 288 and 384 trials per participant).

#### Model

We used a bounded evidence accumulation model to fit the choices and the confidence about motion direction. In the model, these reports are based on the state of accumulated motion evidence (decision variable, *e*) at the time $\left(t_{e}\right)$ that a bound (at ±A) is reached or when the stimulus ends. The decision variable evolves according to 1-dimensional process of Brownian motion plus deterministic drift. In small time steps Δt, the state of the decision variable is updated based on the sum of terms representing a deterministic drift and stochastic diffusion. The drift term is given by κcΔt, where *c* is the signed motion coherence and κ is parameter which determines the signal-to-noise ratio. The diffusion term follows a normal distribution with a standard deviation of $\sqrt{\Delta t}$, such that the variance at t=1s (absent bounds) would equal 1. This is not a free parameter, since for any other value we can obtain an equivalent model by scaling κ and *A* appropriately (Palmer et al., 2005, Shadlen et al., 2006).

The model assumes that the decision maker has access to the state of the decision variable *e* and the decision time $t_{e}$. In the Bayesian model, the reports of choice and confidence are based on the posterior probability over motion direction, $p\left(d|e^{\left(i\right)},t_{e}^{\left(i\right)},E^{\left(i-1\right)}\right)$, and the belief report is based on the posterior probability over base rate, $p\left(B|e^{\left(i\right)},t_{e}^{\left(i\right)},E^{\left(i-1\right)}\right)$. In these expressions, *d* is the motion direction, *B* is the base rate of the block, and *i* is the trial number. $E^{\left(i\right)}$ is the tuple of all observations of *e* and $t_{e}$ up to trial *i* within a block: $E^{\left(i-1\right)}\equiv {\left\{e,t_{e}\right\}}_{1\text{..}i-1}$.

The posterior over the two possible directions of motion, $p\left(d|e^{\left(i\right)},t_{e}^{\left(i\right)},E^{\left(i-1\right)}\right)$, can be obtained by Bayes rule:

(3) $$p\left(d|e^{\left(i\right)},t_{e}^{\left(i\right)},E^{\left(i-1\right)}\right)=\frac{p\left(e^{\left(i\right)},t_{e}^{\left(i\right)}|d,E^{\left(i-1\right)}\right)p\left(d|E^{\left(i-1\right)}\right)}{p\left(e^{\left(i\right)},t_{e}^{\left(i\right)}|E^{\left(i-1\right)}\right)}$$

Which simplifies to:

(4) $$p\left(d|e^{\left(i\right)},t_{e}^{\left(i\right)},E^{\left(i-1\right)}\right)\propto p\left(e^{\left(i\right)},t_{e}^{\left(i\right)}|d\right)\sum_{B}p\left(d|B\right)p\left(B|E^{\left(i-1\right)}\right)$$

where the constant of proportionality is such that the sum of the probabilities of right and left equals 1. The sum on the right side of expression 4 is over the six possible values of *B*.

The likelihood in expression 4 can be computed by marginalizing over motion coherence (*c*):

(5) $$p\left(e^{\left(i\right)},t_{e}^{\left(i\right)}|d\right)=\sum_{c}p\left(e^{\left(i\right)},t_{e}^{\left(i\right)}|c,d\right)p\left(c|d\right)$$

where p(c|d) is a uniform distribution over the coherence values that are compatible with motion direction *d*, and $p\left(e^{\left(i\right)},t_{e}^{\left(i\right)}|c,d\right)$ is obtained from the numerical solutions to the Fokker-Planck equation, which governs the bounded drift-diffusion (Kiani and Shadlen, 2009).

By Bayes rule, the likelihood $p\left(e^{\left(i\right)},t_{e}^{\left(i\right)}|d\right)$ is proportional to the posterior, $p_{u}\left(d|e^{\left(i\right)},t_{e}^{\left(i\right)}\right)$, under a neutral prior. Further, p(d|B) is equal to *B* if *d* is rightward, and to (1−B) if *d* is leftward. These substitutions lead to the expression in Figure 3B.

The choice and the confidence about motion direction are derived from the posterior $p\left(d|e^{\left(i\right)},t_{e}^{\left(i\right)},E^{\left(i-1\right)}\right)$. The decision maker chooses rightward if $p\left(d=R|e^{\left(i\right)},t_{e}^{\left(i\right)},E^{\left(i-1\right)}\right)>0.5$ and chooses leftward otherwise.

The belief is obtained from the posterior over base rate, $p\left(B|e^{\left(i\right)},t_{e}^{\left(i\right)},E^{\left(i-1\right)}\right)$. By Bayes rule:

**Figure:**
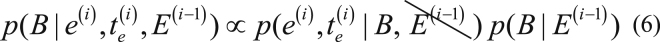

where the constant of proportionality is such that

(7) $$\sum_{B}p\left(B|e^{\left(i\right)},t_{e}^{\left(i\right)},E^{\left(i-1\right)}\right)=1$$

and

(8) $$p\left(e^{\left(i\right)},t_{e}^{\left(i\right)}|B\right)=p\left(e^{\left(i\right)},t_{e}^{\left(i\right)}|R\right)\underset{B}{\underset{︸}{p\left(R|B\right)}}+p\left(e^{\left(i\right)},t_{e}^{\left(i\right)}|L\right)\underset{1-B}{\underset{︸}{p\left(L|B\right)}}$$

The strikethrough in Equation 6 serves as a reminder that $E^{\left(i-1\right)}$ only affects the probability of the evidence through *B*. Again, noting that $p\left(e^{\left(i\right)},t_{e}^{\left(i\right)}|d\right)$ is proportional to the confidence under a neutral prior, $p_{u}\left(d|e^{\left(i\right)},t_{e}^{\left(i\right)}\right)$, we obtain the expression in Figure 3C.

These calculations are iterated for the subsequent trials in the block, where the posterior after one trial becomes the prior for the next one:

(9) $$p\left(B|E^{\left(i\right)}\right)\equiv p\left(B|e^{\left(i\right)},t_{e}^{\left(i\right)},E^{\left(i-1\right)}\right)$$

The Bayesian, Choice-only and Choice-confidence models differ only on the way in which p(B) is updated from one trial to the next (see Equation 1 of the main text).

#### Model Fitting

The model was fit to each subject’s choice and confidence report on each trial. Each trial supplies a left or right choice and a confidence rating, which we transformed into a common monotonic scale (see below). For fitting, we categorized the confidence ratings as either high or low. This allowed us to treat each confidence report as a binary variable with likelihood comparable to the choice. For each subject, the lowest 30% of the confidence reports were considered low confidence. The cutoff seemed sensible given (i) the way confidence reports cluster in the scale (Figure S2), (ii) that it approximates the proportion of low confidence reports in a similar task in which subjects categorized confidence into low or high (van den Berg et al., 2016), and (iii) that changing this value within a reasonable range does not alter the results reported here.

We performed maximum-likelihood fits of the model parameters θ separately for each subject:

(10) $$\hat{\theta }=\underset{\theta }{\text{argmax}}\left(\sum_{j=1}^{M}\sum_{i=1}^{N_{j}}log\left(p\left({\text{choice}}_{j}^{\left(i\right)},{\text{conf}}_{j}^{\left(i\right)}|c_{j}^{\left(i\right)},T_{j}^{\left(i\right)},E_{j}^{\left(i-1\right)},\theta \right)\right)\right)$$

where $N_{j}$ is the number of trials in block *j*, *M* is the number of blocks, choice and conf are the chosen alternative (left/right) and the category of the confidence report (high/low) on trial *i* in block *j*. Note that information from the previous trials in the block affects the current one through the distribution over base rate $p\left(B|E_{j}^{\left(i-1\right)}\right)$. The joint distribution over choice and confidence is calculated by marginalizing over the values of *e* and $t_{e}$ that are consistent with the report. Note for simplicity, from here on, we drop the subscript notation for the block. For instance, the probability of a rightward choice of low confidence is calculated as:

(11) $$p\left(R,\text{low}|c^{\left(i\right)},T^{\left(i\right)},E^{\left(i-1\right)},\theta \right)=\iint p\left(e,t_{e}|c^{\left(i\right)},T^{\left(i\right)},\theta \right)1_{0.5<p\left(R|e,t_{e},E^{\left(i-1\right)},\theta \right)<\phi }de\;dt_{e}$$

where $1_{x}$ is an indicator function that evaluates to 1 when *x* is true and to zero otherwise, and ϕ is a fitted parameter—a criterion on probability correct that separates high from low confidence responses. The indicator function restricts the integration to the range of values of *e* and $t_{e}$ for which the probability of rightward motion is higher that 0.5 but lower than ϕ.

For the first trial in a block, because the subject knows the six possible values that the bias can take and that they are equally likely, the initial distribution over bias $p_{0}\left(B\right)$ should be uniform on these discrete values. However, we assumed that $p_{0}\left(B\right)$ could be non-uniform. The distribution $p_{0}\left(B\right)$ is fully specified by two parameters, because there are six possible values that *B* can take and we assume that the distribution is symmetric around 0.5. For fitting, the weight of the two values closer to 0.5 was set to 1 (before normalizing the probability distribution), and the other two weights, $\omega_{1}$ (for the two intermediate value of *B*) and $\omega_{2}$ (for the highest and lowest values of *B*), were fit to the data. The best-fitting parameters for each subject are shown in Table S2 (see also Figure S6).

To update the probability distribution over *B* from one trial to the next, we need to know the ratio of the likelihoods $p\left(e^{\left(i\right)},t_{e}^{\left(i\right)}|R\right)/p\left(e^{\left(i\right)},t_{e}^{\left(i\right)}|L\right)$, or equivalently, the ratio of the confidence in rightward and leftward choices under a neutral prior, $p_{u}(R|e^{(i)},t_{e}^{(i)})/p_{u}(L|e^{(i)},t_{e}^{(i)})$. These likelihoods depend on state of accumulated evidence *e* and the decision time $t_{e}$ of each trial, which are not available to the experimenter. However, we can use Bayes rule to derive the ratio from the transformed confidence reports and the distribution over base rates from the previous trial.

On trials in which subjects chose rightward, confidence is equal to $p\left(R|e^{\left(i\right)},t_{e}^{\left(i\right)},E^{\left(i-1\right)}\right)$ which by Bayes rule is:

**Figure:**
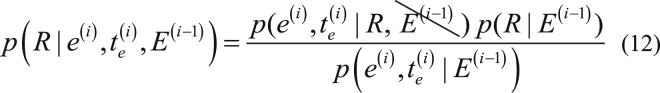

where the strike-through makes explicit supervenience (i.e., given direction, the evidence from previous trials is irrelevant to the likelihood). Similarly,

(13) $$p\left(L|e^{\left(i\right)},t_{e}^{\left(i\right)},E^{\left(i-1\right)}\right)=\frac{p\left(e^{\left(i\right)},t_{e}^{\left(i\right)}|L\right)p\left(L|E^{\left(i-1\right)}\right)}{p\left(e^{\left(i\right)},t_{e}^{\left(i\right)}|E^{\left(i-1\right)}\right)}=1-p\left(R|e^{\left(i\right)},t_{e}^{\left(i\right)},E^{\left(i-1\right)}\right)$$

Then the ratio of the likelihoods can be derived from the confidence reported by the subject and the prior expectation over direction of motion that is updated over trials:

(14) $$\frac{p\left(e^{\left(i\right)},t_{e}^{\left(i\right)}|R\right)}{p\left(e^{\left(i\right)},t_{e}^{\left(i\right)}|L\right)}=\left(\frac{p\left(R|e^{\left(i\right)},t_{e}^{\left(i\right)},E^{\left(i-1\right)}\right)}{1-p\left(R|e^{\left(i\right)},t_{e}^{\left(i\right)},E^{\left(i-1\right)}\right)}\right)\left(\frac{1-p\left(R|E^{\left(i-1\right)}\right)}{p\left(R|E^{\left(i-1\right)}\right)}\right)$$

Because we cannot assume that the numerical confidence ratings directly reflect the internal estimates of the probability of being correct, we applied a monotonic transformation to the ratings, which captures their expected distribution under the three models. To transform the reported confidence into the posterior probability that *d* is the correct choice, $p\left(d|e^{\left(i\right)},t_{e}^{\left(i\right)},E^{\left(i-1\right)}\right)$, we assumed that $p\left(d|e^{\left(i\right)},t_{e}^{\left(i\right)},E^{\left(i-1\right)}\right)$ is a monotonic but potentially nonlinear function of the reported confidence. We derived this transformation empirically. Each behavioral trial was assigned a value of probability correct from simulations of the model. The assignment preserves the rank-order of the confidence reports; for instance, the behavioral trial with highest confidence is assigned the highest value of confidence produced by the model. Note that values of *probability correct* obtained from simulations of the model depend on the model parameters. Therefore the transformation of the confidence ratings must be established iteratively during model fitting. Importantly, the transformation does not affect the high/low designation because it preserves the order of the raw confidence reports. It does affect the model predictions because it affects counterfactual confidence, hence the update of p(B).

The parameter-free transformation of confidence ratings to probability correct (shown in Figure S7A) ensures that the confidence distributions of the model and the data are identical. Therefore, we can compare the analog confidence reports between model and data as a function of the variables of interest: choice accuracy, motion strength, and base rate (Figure S7B). We also used the transformed reports in the Bayesian and Choice-confidence models. The conclusions of our study would not change had we used the raw confidence reports, although without the transformation the model could not explain the idiosyncrasies in the confidence ratings (e.g., the over and under confidence displayed by different participants; Figure S7C).

The Bayesian, Choice-only and Choice-confidence models use the same five parameters: κ and *A* (drift-diffusion); ϕ (confidence binarization); $\omega_{1}$ and $\omega_{2}$ (initial prior over base rate). We used the Bayesian Adaptive Direct Search (BADS) method to find the maximum-likelihood parameters (Acerbi and Ma, 2017). We used different initial starting points (N = 30) to reduce the risk of finding local maxima.

From the best fitting model parameters, we predicted the participants belief reports using the posterior $p\left(B|e^{\left(i\right)},t_{e}^{\left(i\right)},E^{\left(i-1\right)}\right)$. The belief that the block is biased to the right is given by

(15) $$\text{belief}\equiv \sum_{B>0.5}p\left(B|e^{\left(i\right)},t_{e}^{\left(i\right)},E^{\left(i-1\right)}\right)$$

In the empirical model (Figure 5D), we parameterized $\nu_{d}$ (from expression 1) as a logistic function with five parameters:

(16) $$logit\left[\nu_{d}\right]=\beta_{0}+\beta_{1}\text{conf}+\beta_{2}p\left(d|E^{\left(i-1\right)}\right)+\beta_{3}p\left(d|E^{\left(i-1\right)}\right)\text{conf}+\beta_{4}\frac{\text{conf}}{p\left(d|E^{\left(i-1\right)}\right)}$$

where ’conf’ is the transformed confidence. The β are fit simultaneously with the other 5 parameters of the model. The mapping from confidence and motion expectation to $\nu_{d}$ for the best-fitting model is shown in Figure 5D. Similar results were obtained using the raw confidence ratings instead of the transformed confidence in Equation 16.

The model predictions displayed in Figures 4, 6, 7, and 8 were obtained from simulations using the fitted parameters. Predictions are based on 200 simulations of the full experiment, using the same sequence of trials (motion strength, direction, and duration) as those performed by the participants. To produce the smooth lines in Figure 4 at interpolated values, we simulated each trial repeatedly with a fine grid of motion strengths (Figures 4A and 4D), and stimulus durations (Figures 4B and 4E). Each interpolated point requires simulation of the entire experiment.

We considered two models to explain the apparent lag between reported belief and model predictions, evident in Figure 6B. In the first, the lag only affects the expression of the report, analogous to capacitor charge, in accordance with the difference equation,

(17) $$f_{i}=f_{i-1}+\left(b_{i}-f_{i-1}\right)\left(1-\alpha \right)$$

where $f_{i}$ is the belief reported on trial *i* and $b_{i}$ is the belief that would have been reported if there were no lag in the belief reports (Equation 2). The time constant, α, was fit to minimize the mean-squared error between model and data (Figure 8B).

We also explored an alternative non-Bayesian model in which the lag affects the update of p(B), and thus choice and confidence. In this alternative model the distributions over base rate for trial *i*, $p_{i}\left(B\right)$, is a weighted average of the distribution over base rates from the previous trial, $p_{i-1}\left(B\right)$, and the distribution that would result if p(B) were updated in a Bayesian manner, $p_{i}^{b}\left(B\right)$,

(18) $$p_{i}\left(B\right)=p_{i-1}\left(B\right)+\left(p_{i}^{b}\left(B\right)-p_{i-1}\left(B\right)\right)\left(1-\alpha \right)$$

We fit this model to the choice and confidence data, refitting all parameters of the Bayesian model plus the α parameter. We also use this alternative implementation of lag in the Choice-only and Choice-confidence models, replacing $p^{b}\left(B\right)$ in Equation 18 by the value of p(B) of the matching model without lag.

#### Data Analysis

We used linear and logistic regression models to evaluate the influence of the base rate, motion strength, and viewing duration, on choice, confidence, and belief. We used t tests (using the parameter estimates and its standard error) to evaluate the null hypothesis that a single coefficient of a linear regression model is zero. For logistic regression models, we used likelihood-ratio tests for nested models to evaluate the null hypothesis that one or more of the regression coefficients were equal to zero. Unless otherwise indicated, we incorporated data from the three participants in all regression models using indicator variables.

The logistic regression model used to examine the effect of base rate on choice (solid lines of Figure 2A) is:

(19) $$logit\left[p_{+}\right]=\beta_{1}Tc+\sum_{i=1}^{n_{B}}\left(\beta_{2,i}c+\beta_{3,i}\right)I_{B_{i}}+\sum_{j=1}^{n_{S}-1}\beta_{4,j}I_{S_{j}}$$

where $p_{+}$ is the probability of a rightward choice, *c* is the motion coherence and *T* is the stimulus duration. The subscripted indicator variables *I* are over base rate *B* and subject S. For instance, $I_{B_{i}}$ is equal to 1 for trials from blocks with base rate $B_{i}$, and zero otherwise. The second subscripts of the β parameters indicate separate parameters fit over the indexed categorical variables. $n_{B}$ and $n_{S}$ are the number of unique base rates and the number of subjects respectively. Note that the $\beta_{4}$ coefficient associated with the last subject is omitted from the equation to avoid redundant parameterization (singular design matrix). The inset in Figure 2A shows the $\beta_{3,i}$ coefficients and associated standard errors obtained from the logistic fits using combined data from all subjects.

To examine the influence of base rate and motion strength on confidence, we used the following linear regression model, fit using correct decisions only:

(20) $$\text{conf}=\beta_{1}\left|c\right|+\beta_{2}B_{sr}+\beta_{3}h+\sum_{i=1}^{n_{S}}\beta_{4,i}I_{S_{i}}$$

where conf is the confidence report (0.5-1 scale), $B_{sr}$ is the base rate relative to the choice (*B* for rightward and 1−B for leftward choices), and *h* is the choice (left or right) to account for potential differences in confidence due to chosen side.

The regression model used to evaluate how accuracy depends on motion strength, motion viewing duration, trial number, and base rate (Figure 4C) is:

(21) $$logit\left[p_{c}\right]=\beta_{1}\left|c\right|+\beta_{2}T+\sum_{i=1}^{n_{S}-1}\beta_{3,i}I_{S_{i}}+\sum_{j=1}^{n_{S}}\beta_{4,j}\left|c\right|I_{S_{j}}+\sum_{k=1}^{n_{tr}}\left(\beta_{5,k}+\beta_{6,k}B_{s}\right)I_{N_{k}}$$

where $p_{c}$ is the probability of a correct response, *N* is the trial number, $n_{t}r$ is the maximum number of trials in a block and $B_{s}$ is the informativeness of the base rate (i.e., either 0.6, 0.8, or 1). Figure 4C (top) shows the $\beta_{6}$ coefficients.

A similar logistic regression model was fit to confidence (Figure 4F):

(22) $$logit\left[p_{h}\right]=\beta_{1}\left|c\right|+\beta_{2}T+\beta_{3}h+\beta_{4}a+\sum_{i=1}^{n_{S}-1}\left(\beta_{5,i}+\beta_{6,i}|c|\right)I_{S_{i}}+\sum_{j=1}^{n_{S}}\left(\beta_{7,j}|c|a\right)I_{S_{j}}+\sum_{k=1}^{n_{tr}}\left(\beta_{8,k}+\beta_{9,k}B_{sr}\right)I_{N_{k}}$$

where $p_{h}$ is the probability of a high-confidence report, *h* is the choice (left or right) to account for potential differences in confidence due to chosen side, and *a* is the accuracy of the response (0 or 1 for incorrect and correct decisions respectively). Figure 4F (bottom) shows the $\beta_{9}$ coefficients. The extra terms are included as potential confounders.

The same regressions were used for both data and model in Figures 4C and 4F. For the model, we conducted the logistic regressions independently for 200 simulations the model and then averaged the regression coefficients. The standard errors (gray shading) are the standard deviation of the coefficients from the 200 simulations. The model was evaluated on the same sequence of trials as the participants.

To test the influence of motion strength on ΔBelief (Figure S5), we fit the following linear regression model:

(23) $$\text{ΔBelief}=\beta_{1}\left|c\right|+\beta_{2}T+\beta_{3}{\text{Belief}}_{c}+\sum_{i=1}^{n_{S}}\beta_{4,i}I_{S_{i}}$$

where ΔBelief is the difference between the belief reported in the current trial and the belief reported in the previous trial (or the difference from ½ for the first trial of the block). To simplify the regression model, ΔBelief was multiplied by −1 when the subject chose the leftward direction of motion. Therefore a positive ΔBelief indicates a change in the direction of the choice. Accordingly, ${\text{Belief}}_{c}$ is the belief in the current trial (see Figure 6) relative to the actual choice (i.e., ${\text{Belief}}_{c}$ is equal to the reported belief for rightward choices, and to one minus the reported belief for leftward choices). As in Figure S5, only correct trials were used for the regression model.

We used the following logistic regression model to test the influence of the block’s base rate on the choices for 0% coherence trials:

(24) $$logit\left[p_{\text{consistent}}\right]=\beta_{1}B_{s}+\beta_{2}T+\sum_{i=1}^{n_{S}}\beta_{3,i}I_{S_{i}}$$

where $p_{\text{consistent}}$ is the probability that the choice is made in the direction considered most likely by the subject, that is, consistent with the belief reported on the previous trial. In the regression analysis we only included trials of 0% motion strength and those in which the belief in the previous trial was either lower than 0.05 or higher than 0.95.

In the analysis of Figure 8, we used the MSE between the reported belief and the model prediction to compare the goodness-of-fit of the models with and without lag. We evaluated the significance of the difference in the MSE by simulating each model 200 times, and defining the p value as the proportion of times in which the model with lag had a lower MSE than the Bayesian model.

### Data and Software Availability

Data and code are available at https://github.com/arielzylberberg. Behavioral data is also available at https://dx.doi.org/10.17632/vzhs4m5573.1.

## Supplemental Information

Document S1. Figures S1–S8 and Tables S1 and S2

Document S2. Article plus Supplemental Information
