# Supplementary material for: Counterfactual Reasoning Underlies the Learning of Priors in Decision Making
Source: Neuron. 2018 Sep 5;99(5):1083–1097.e6. doi: 10.1016/j.neuron.2018.07.035 (PMC6127036; doi:10.1016/j.neuron.2018.07.035)
Supplement: Document S1. Figures S1–S8 and Tables S1 and S2 [file mmc1.pdf]

Neuron, Volume *99*

Supplemental Information

Counterfactual Reasoning Underlies the Learning of Priors in Decision Making

Ariel Zylberberg, Daniel M. Wolpert, and Michael N. Shadlen

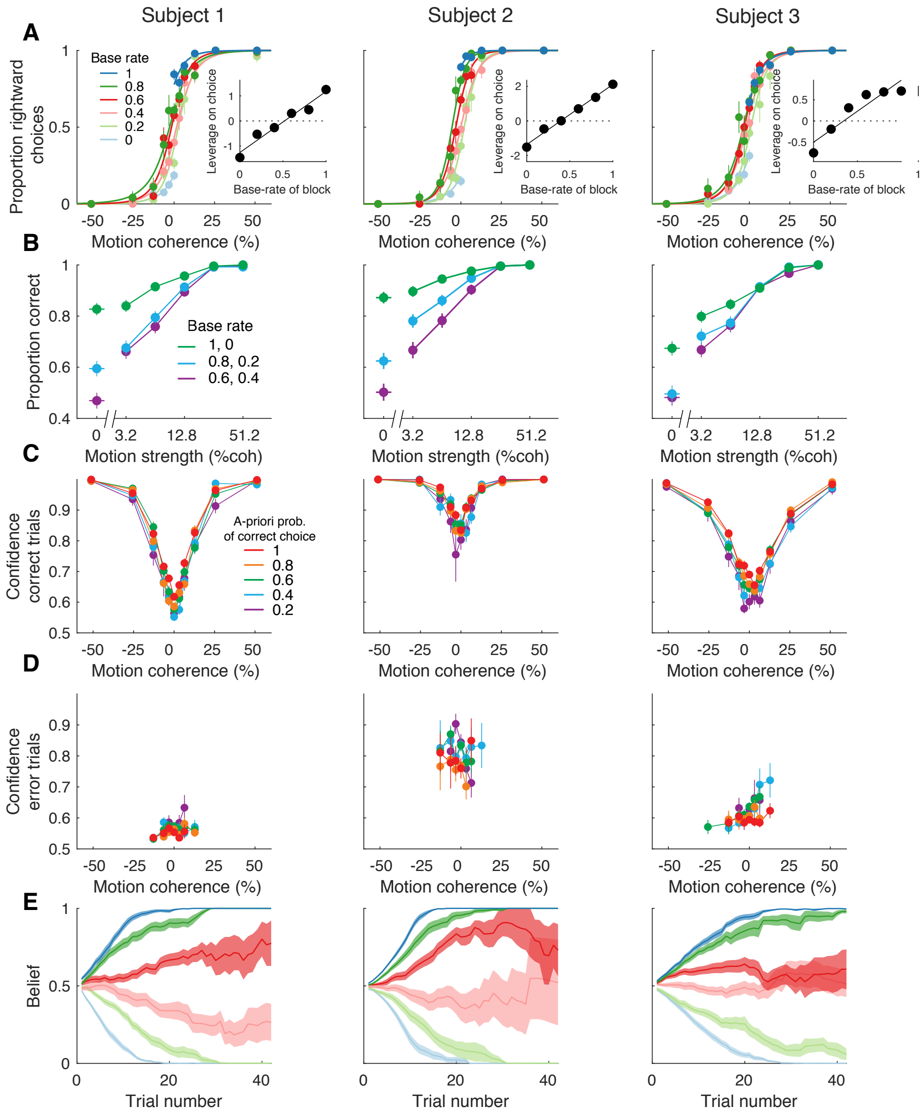

Figure S1. Behavioral influence of the block's base rate. Related to Figure 2. Same graphs and data as in Figure 2, but shown separately for each participant (columns). Rows A,B,C,E correspond to panels A-D of Figure 2. Panel D shows the average confidence ratings for error trials split by the *a priori* probability correct of the block. Same color convention as panel C. For example, the points in orange comprise trials in which the subject erroneously chose left in blocks containing 80% rightward motion, or erroneously chose right in blocks containing 80% leftward motion. Points comprising less than 5 trials are not shown.

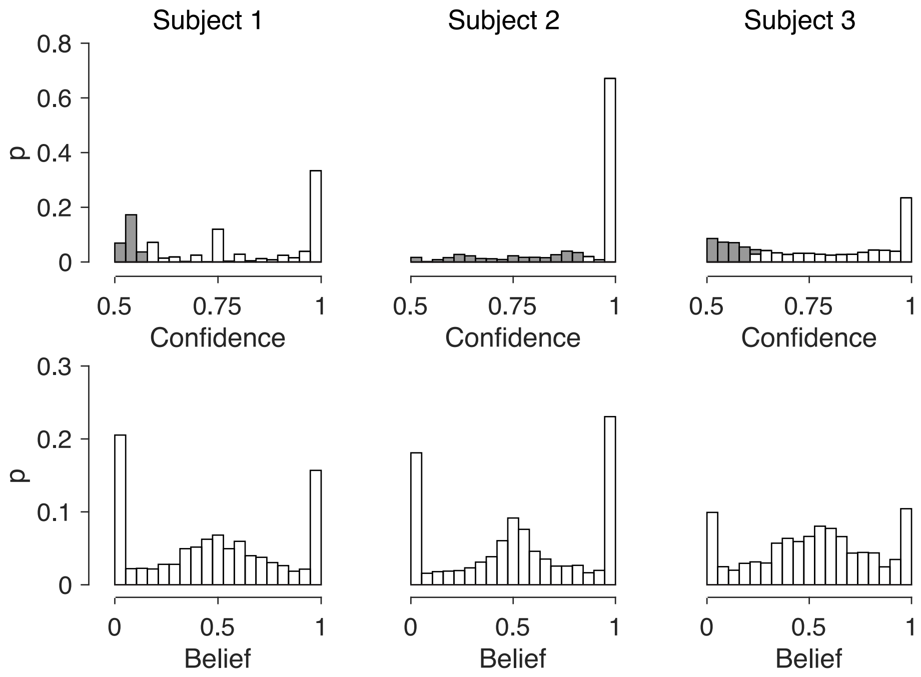

Figure S2. Distributions of confidence and belief reports. Related to Figure 2. *Top row*, distributions of confidence reports for the 3 participants. Shading indicates the trials below the 30th percentile, which we designate "low confidence". *Bottom row*, distributions of belief reports for the 3 participants.

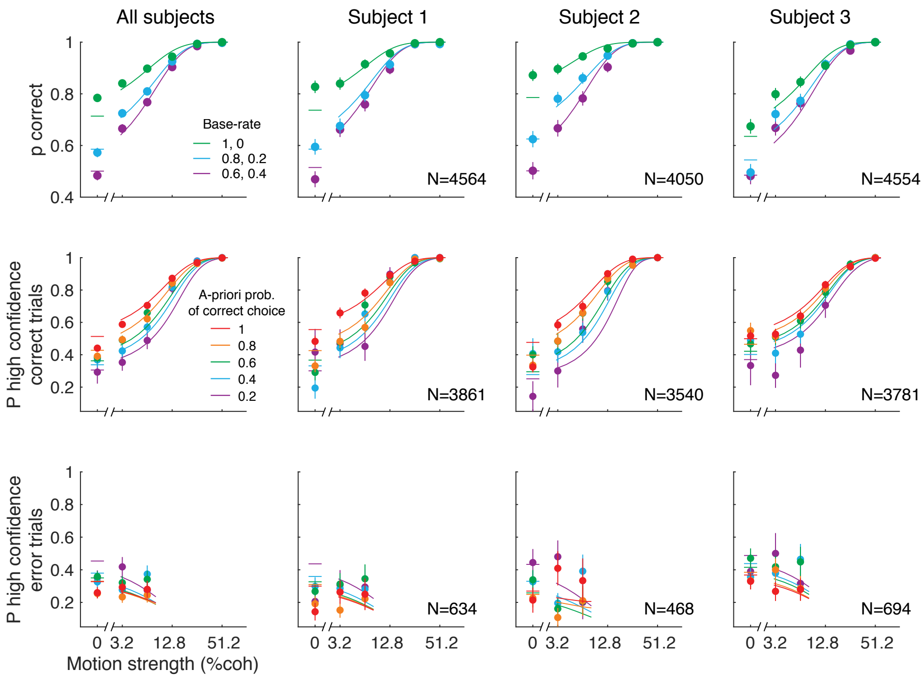

Figure S3. Fits of the Bayesian model to choice and confidence, combined and by subject. Related to Figure 4. Left column shows combined data from all subjects, and columns 2-4 show the fits for the individual subjects. *Top*, Combined data is a reproduction of Figure 4A. *Middle*, Combined data is a reproduction of Figure 4D. *Bottom*, Same as middle row, but for the error trials. Fits (solid lines) were obtained using all trials, so dominated by the correct choices (errors constitute 13-17% of trials). Points comprising less than 10 trials are not shown.

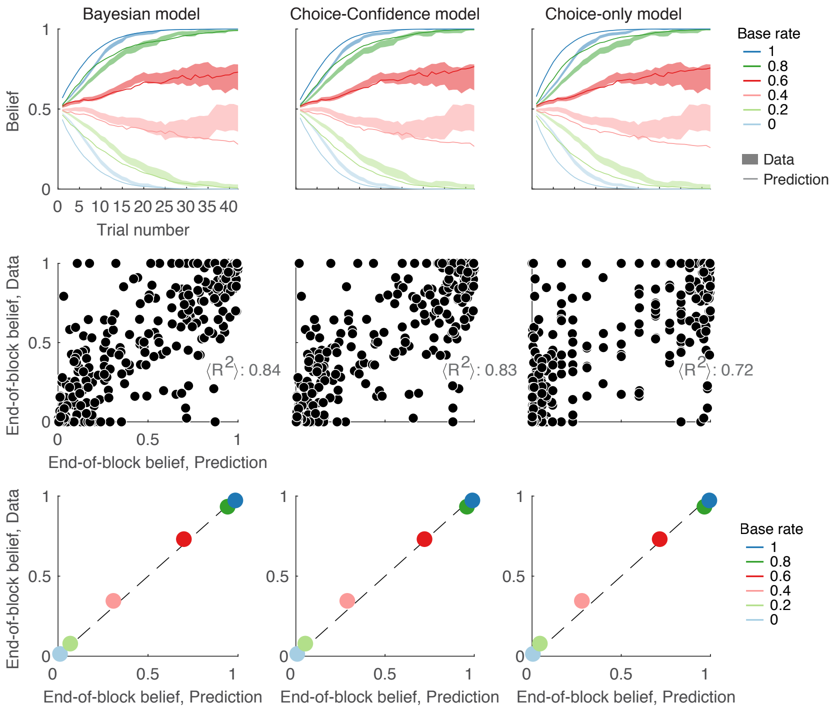

Figure S4. Belief predictions for the three models. Related to Figure 6. Same analyses as in figure 6B-D, for the belief predictions of the three models: Bayesian (first column), Choice-confidence (second column) and Choice-only (third column) models.

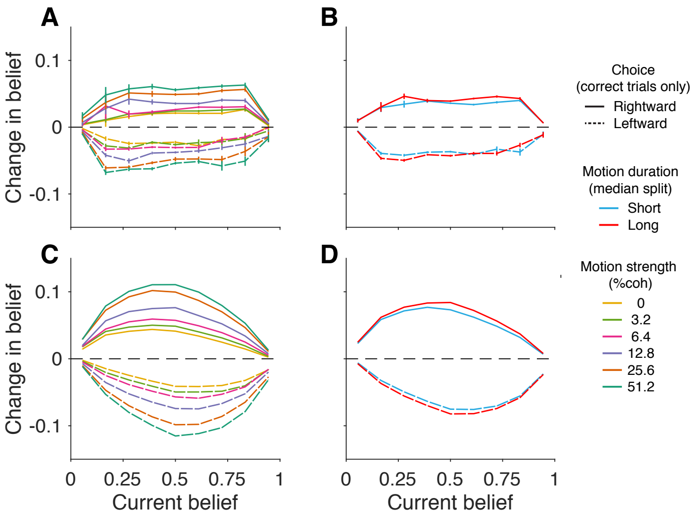

Figure S5. Change in belief depends on current belief, choice, motion strength and duration. Related to Figure 6. All panels depict the average change in the belief, from one trial to the next, that the base rate assigned to the block favors rightward. Only correct trials are included in this analysis. **(A)** Data split by choice (line style) and motion strength (color). For each coherence and for the two choices, the belief increased when participants chose rightward motion and decreased when they chose leftward motion. Further, because the scale is bounded, the changes approach zero at the extremes. More interestingly, for the same level of the current belief, stronger motion led to larger changes in belief (Eq. 23; $p < 10^{-8}$, t-test, $H_0 : \beta_1 = 0$). The traces were obtained by grouping trials in 9 equally-spaced bins of belief. The error bars indicate s.e.. **(B)** Data split by choice and median duration (color), combining all motion strengths. Longer durations were associated with larger changes in belief (Eq. 23; $p < 10^{-8}$, t-test, $H_0 : \beta_2 = 0$). **(C & D)** Simulations of the Bayesian model; same conventions as A & B. A prediction of the Bayesian model is that stronger motion, which is associated with higher confidence on average, should lead to a larger change in the belief that the block is biased to the right or left. The predicted changes are larger than observed. One factor that contributes to this mismatch is that participants' reports appeared to lag behind their internal representation of belief (see main text). Importantly, and regardless of the difference in absolute magnitude, the relationship between motion strength and change in belief is incompatible with models in which only choice—and not the certainty of the choice—informs the revision of belief. For the model predictions, we grouped trials from 200 simulations of the full experiment.

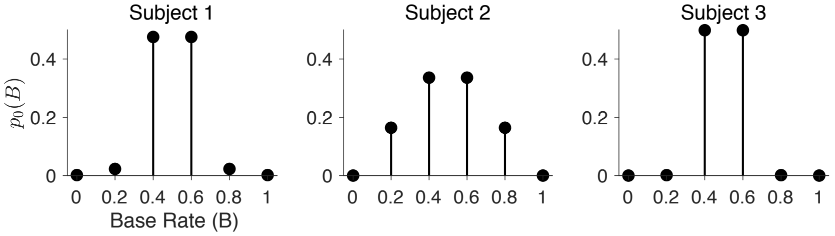

Figure S6. Initial prior probability distributions of base rate, $p_0(B)$, for the three participants. Related to STAR Methods. Although the base rate for the block is chosen from a uniform distribution over the six values $B \in [0, 0.2, 0.4, 0.6, 0.8, 1]$, the model allows for the possibility that the participant does not represent this prior veridically. The graphs show the probability distributions over base rate at the beginning of each block, obtained from the model fit to the choice and confidence. These distributions are described by 2 parameters under the assumption of symmetry about 0.5. Subjects did not report their initial belief, and the belief reports played no role in the fit. All three participants assigned higher probability to bias values close to 0.5.

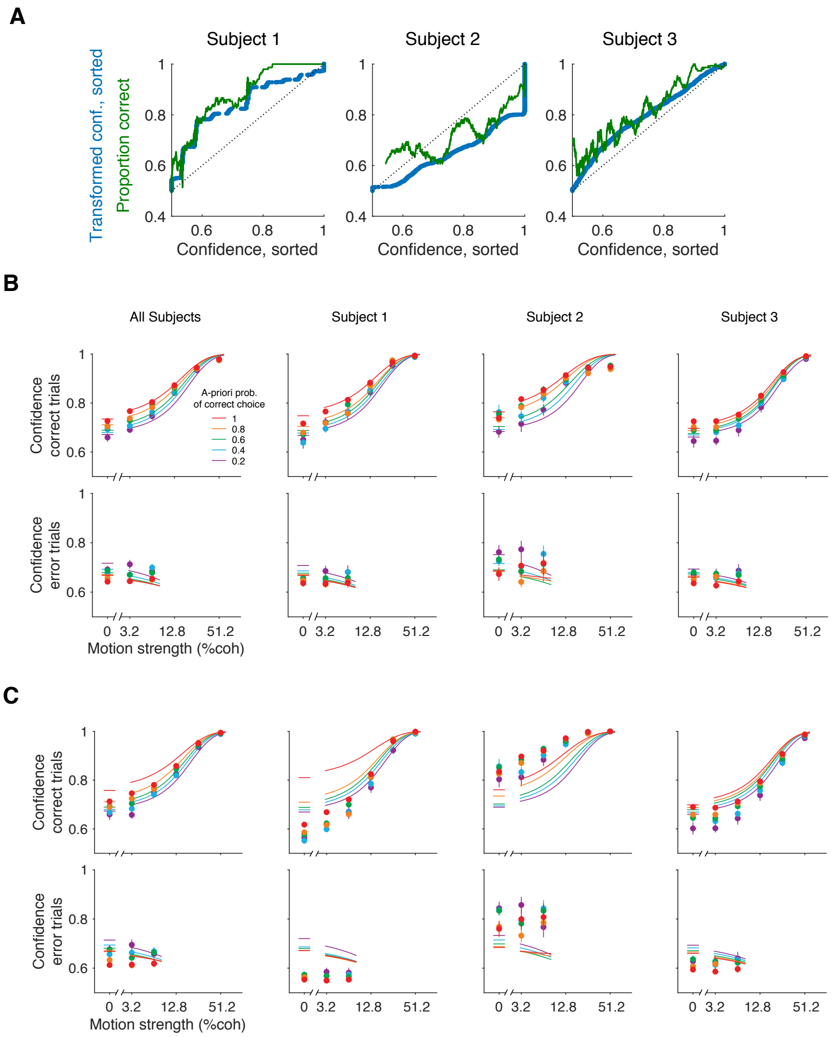

Figure S7. Remapping of reported confidence to a common probability scale. Related to STAR Methods. As shown in Supplementary Figure S2, each of the participants used the confidence rating scale differently. We assumed these reports are monotonically related to the actual confidence—that is, the probability that the choice made was correct. The method to achieve this transformation (i.e., remapping) is explained in Methods. This figure supports the use of this transformation. (A) Comparison of raw and remapped confidence reports. The blue trace are ordered pairs of the sorted raw confidence reports and their transformed value (no smoothing). The green trace show the corresponding proportion correct (running average, N=150). The match between blue and green curves indicates that our transformation of the confidence ratings roughly approximates the proportion of correct responses for each level of confidence. (B) Average confidence (remapped) in groups of trials determined by combinations of motion strength (abscissa), bias strength (colors) and accuracy (top and bottom rows). The agreement between model and data indicates that the use of remapping allows to explain not only the confidence ratings categorized into high/low, but the actual analog values. (C) As in B, but for a model without remapping (i.e., one in which we take the confidence ratings as veridical reports of probability correct).

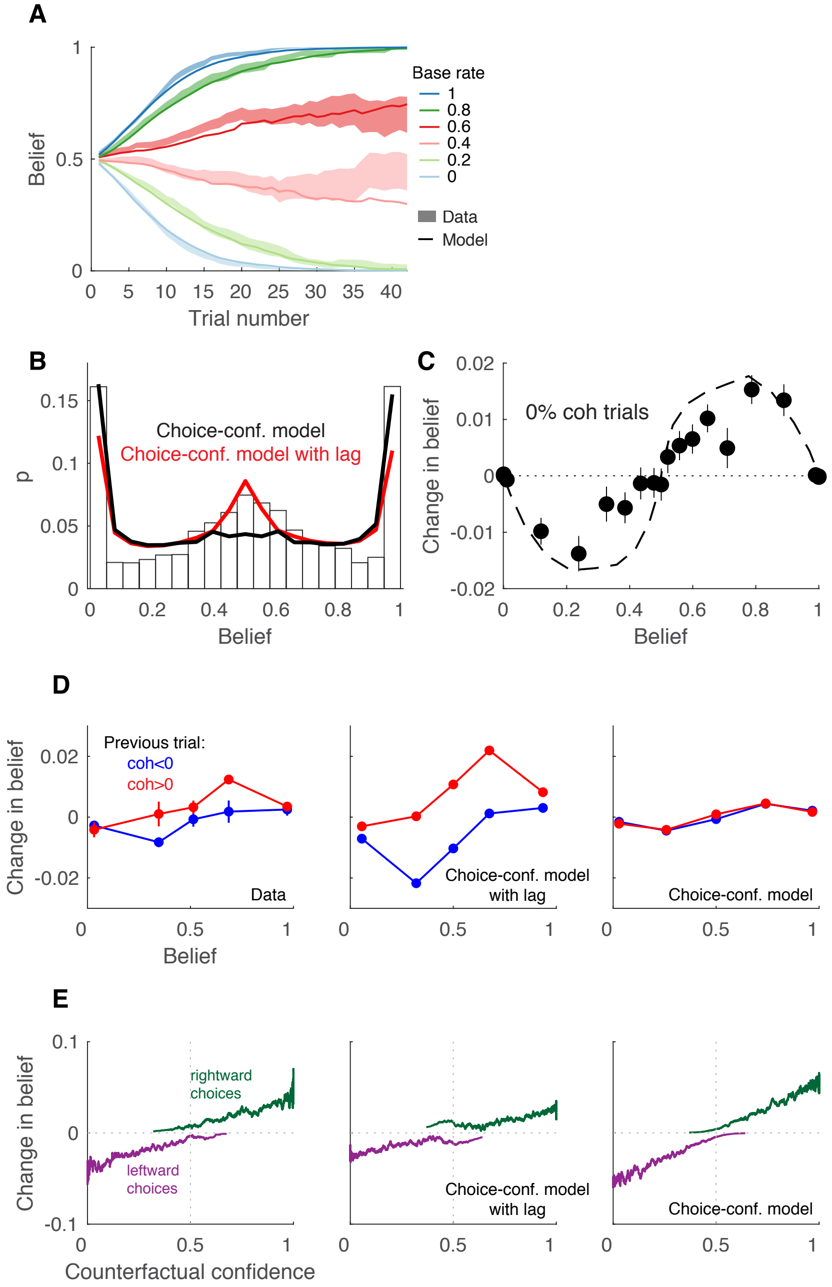

Figure S8. Choice-confidence model with lag. Related to Figure 8. Same analyses as in Figure 8, but for simulations of the Choice-confidence model.

	Participants			
	**S1**	**S2**	**S3**	**Combined**
Choice-only model	29	39	2	70
Choice-confidence model	29	43	3	75
Bayesian model	**0**	**0**	**0**	**0**

Table S1. Differences in log-likelihood relative to best model for each subject. The best model for each subject is highlighted in bold. The log-likelihood for the Bayesian model are -3142, -2612 and -3506 respectively for the three participants. Related to STAR Methods.

.

		Participants		
		**S1**	**S2**	**S3**
κ	signal-to-noise	19.42	23.27	16.67
A	bound height	1.55	1.12	3.45
ω_1	weight for 2nd and 5th elements of $p_0(B)$	0.05	0.49	0.003
ω_2	weight for 1st and 6th elements of $p_0(B)$	0.004	0	0
ϕ	high/low confidence separatrix	0.72	0.78	0.68

Table S2. Best-fit parameters of the Bayesian model. Related to STAR Methods.
